# Recent Advances in Ligand Design and Engineering in Lead Halide Perovskite Nanocrystals

**DOI:** 10.1002/advs.202100214

**Published:** 2021-05-05

**Authors:** Katie Hills‐Kimball, Hanjun Yang, Tong Cai, Junyu Wang, Ou Chen

**Affiliations:** ^1^ Department of Chemistry Brown University Providence RI 02912 USA

**Keywords:** device integration, ligand characterization, ligand engineering, nanocrystal surface design, optical and optoelectronic properties, perovskite nanocrystals, stability

## Abstract

Lead halide perovskite (LHP) nanocrystals (NCs) have recently garnered enhanced development efforts from research disciplines owing to their superior optical and optoelectronic properties. These materials, however, are unlike conventional quantum dots, because they possess strong ionic character, labile ligand coverage, and overall stability issues. As a result, the system as a whole is highly dynamic and can be affected by slight changes of particle surface environment. Specifically, the surface ligand shell of LHP NCs has proven to play imperative roles throughout the lifetime of a LHP NC. Recent advances in engineering and understanding the roles of surface ligand shells from initial synthesis, through postsynthetic processing and device integration, finally to application performances of colloidal LHP NCs are covered here.

## Graphical abstract

Lead halide perovskite nanocrystals possess labile ligand coverage, yielding a highly dynamic ligand system that can be affected by delicate changes in nanocrystal environment. As such, ligands play imperative roles throughout the lifetime of these ionic nanocrystals. Ligand roles in synthesis, postsynthetic processing, device integration, and application performance of lead halide perovskite nanocrystals are covered.

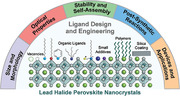

## 1 Introduction

The ever‐growing field of nanomaterials has witnessed births and developments of a wide variety of nanocrystals which have been designed and manipulated to gain material properties that could not be afforded on the bulk scale. Among the heavily studied categories of nanomaterials, semiconductor nanocrystals (NCs), also known as quantum dots (QDs), have shown great promise for a multitude of applications owing to their unique size‐dependent properties through quantum confined electronic structures.^[^ ^1^, ^2^, ^3^, ^4^ ^]^ In addition, due to their small sizes (typically ranging between 5–20  nm), thus high surface‐area‐to‐volume ratios, colloidally synthesized QD NCs are covered with a ligand shell which typically consists of organic molecules possessing functional groups with affinity to the particle surface as well as solvent molecules.^[^ ^1^, ^3^, ^4^, ^5^, ^6^, ^7^, ^8^, ^9^, ^10^, ^11^, ^12^, ^13^, ^14^, ^15^, ^16^ ^]^ As a result, these ligands can effectively passivate the QD surface with one end while extending the other outward into the solution, imparting colloidal dispersibility of the QD NCs.^[^ ^5^, ^16^, ^17^ ^]^ Much beyond offering colloidal stabilities, ligand shells play a series of imperative roles throughout the lifetime of a QD NC. Even before QD nucleation, ligands often coordinate to precursor materials, aiding in solubilization and formation of monomers, as well as influencing the availability of the monomers during the nucleation process.^[^ ^5^, ^16^, ^18^, ^19^ ^]^ Later in the synthesis, ligands act to impact the nucleation, passivate the formed nuclei, balance the QD growth‐dissolution equilibrium, and prevent unwanted Ostwald ripening or further growth of the QD beyond the nanoscale. In all, this can affect the resulting QD NCs’ size, morphology, monodispersity and crystallinity.^[^ ^5^, ^6^, ^18^, ^19^, ^20^, ^21^, ^22^, ^23^, ^24^ ^]^ Postsynthesis, the presence of ligands on the QD surface affirms superior optical and optoelectronic properties, affects heterostructure/composition formation, influences ion exchanges and impurity doping reactions, as well as dictates dispersibility of the QD NCs in any desired solvents.^[^ ^3^, ^11^, ^13^, ^17^, ^18^, ^25^, ^26^, ^27^, ^28^, ^29^, ^30^, ^31^ ^]^ Furthermore, the interparticle ligand–ligand interactions largely impact QD assembly behavior and film formation controllability,^[^ ^14^, ^15^, ^32^, ^33^, ^34^ ^]^ which is crucially important for device integrations in various solid‐state applications.^[^ ^32^, ^35^, ^36^ ^]^ Taken together, ligands play irreplaceable roles in QD NC design, synthesis and application, making them an important area of research ever since the discovery of colloidal QD NCs.

**Figure 1:**
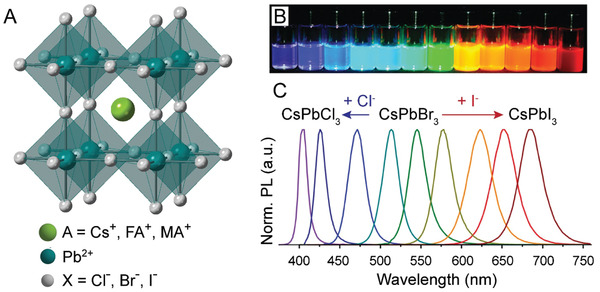
The LHP structure and tunable LHP NC optical properties. A) Schematic representation of the perovskite structure showing one corner‐sharing BX_6_ cuboctahedron housing an “A” cation. B) Pictures of colloidal solutions of perovskite NCs with varying halide compositions demonstrating the NC brightness and wide range of PL emissions and C) selected corresponding PL spectra. B,C) Adapted with permission.^[^ ^43^ ^]^ Copyright 2015, American Chemical Society.

Lead halide perovskite (LHP) NCs are one type of semiconductor QD NCs that have recently increased in popularity by leaps and bounds.^[^ ^37^, ^38^, ^39^, ^40^, ^41^ ^]^ The name “perovskite” was originally used to describe isostructural “ABO_3_” oxide compounds following the initial discovery of the CaTiO_3_ mineral in the Ural Mountains by Lev Perovski and Gustav Rose (1838).^[^ ^42^ ^]^ LHPs specifically, possess an “ABX_3_” formula where the monovalent “A” cation is either an inorganic cation (typically Cs^+^) or a small organic cation (commonly methylammonium (MA^+^) or formamidinium (FA^+^)), “B” is Pb^2+^, and “X” is Cl^–^, Br^–^, or I^–^. In turn, the perovskite structure is constructed of a network of corner sharing ^“^BX_6_^”^ octahedra which house “A” cations at the center of each resulting cuboctahedron (**Figure** **1**A).^[^ ^43^, ^44^ ^]^ Following the first reported synthesis of nanoscale perovskite particles in 2014^[^ ^37^ ^]^ and the subsequent seminal works of the ligand assisted reprecipitation (LARP)^[^ ^45^ ^]^ and hot injection syntheses of LHP NCs,^[^ ^43^ ^]^ thousands of additional publications have followed, each further pushing the field toward better control of these highly ionic NCs with demonstrated superior optoelectronic properties. Specifically, LHP NCs have been shown to possess narrow PL emission linewidths, long diffusion carrier lengths, and a defect tolerant nature, leading to as synthesized, close to unity PL quantum yields (PL QYs).^[^ ^37^, ^38^, ^43^, ^46^ ^]^ Taken together, these properties make LHP NCs suitable for a wide spectrum of applications ranging from solar energy harvesting to new generation displays and lighting.^[^ ^47^, ^48^, ^49^, ^50^, ^51^, ^52^, ^53^, ^54^, ^55^ ^]^

Similar to other semiconducting QD NCs, emission of the LHP NCs can be tuned to cover the entire visible range of the electromagnetic spectrum (Figure 1B,C).^[^ ^43^ ^]^ This is typically done by varying the halide composition, which in turn alters the energy of the bandgap.^[^ ^43^, ^45^, ^56^, ^57^, ^58^, ^59^, ^60^, ^61^, ^62^ ^]^ Compositional tuning has been expanded even further for these materials, as altering other ions in the perovskite structure has been demonstrated to change a variety of NC properties (including absorption edge and PL emission, lifetime decay, PL QY, crystalline growth control, and structural stability), or to add additional dopant properties (like dual emission observed when doping in Cd^2+^, Mn^2+^, or Yb^3+^ for Pb^2+^).^[^ ^63^, ^64^, ^65^, ^66^, ^67^, ^68^, ^69^, ^70^, ^71^, ^72^, ^73^, ^74^, ^75^, ^76^, ^77^ ^]^ Engineering the composition of LHP NCs has been shown to be successful through both synthetic and postsynthetic means, further extending the potential for LHP NCs to be extremely useful and highly adjustable to meet the requirements set by different applications.^[^ ^39^, ^76^, ^78^ ^]^

With the demonstration of superior optoelectronic performance and well‐established methods for property control, LHP NCs have proven themselves as important and promising materials. However, many challenges and problems associated with LHP NCs still exist, which need to be addressed before practical application and commercialization become feasible. For instance, although they are known for their high defect tolerance, LHP NCs are not free of all defects, and typically suffer from halide vacancies resulting in undercoordinated Pb^2+^ and lowered PL QYs, consequently affecting their performances in applications.^[^ ^79^, ^80^ ^]^ Furthermore, LHP NC stability is one of the key challenges that has plagued this field from the very beginning. Extreme NC sensitivity to water, sunlight and air, long‐term storage issues, facile ion exchange reactions limiting the combination of different types of perovskite NCs into inks and devices, etc., have caused tremendous issues in LHP NC developments and implementations.^[^ ^81^, ^82^ ^]^ Accordingly, research into strategies to circumvent some of these issues has been heavily focused on. As such, the design of the organic ligand shell surrounding the LHP NCs represents a method that has been developed to treat surface defects and enhance stability. Ligand engineering has also proven valuable for control over a variety of other properties of LHP NCs, including particle size, morphology, and crystallinity, optical and optoelectronic output, postsynthetic transformation and assembly, and device compatibility and performance.

In considering the ligand shell, additional and unprecedented challenges arise in LHP NC materials that cause them to substantially differ from traditional QDs (e.g., CdSe QDs).^[^ ^80^ ^]^ As a tri‐component crystalline system, LHP NCs are subject to multiple, and different types of defects;^[^ ^83^ ^]^ as highly ionic materials, they have different ligand requirements;^[^ ^80^, ^83^, ^84^ ^]^ and, in some cases, as mixtures of inorganic and organic ions, they possess distinct and more complex surface chemistry.^[^ ^83^ ^]^ Typically, syntheses of LHP NCs rely on using a combination of alkylamines and alkyl carboxylic acids as the ligand shell.^[^ ^37^, ^43^, ^45^, ^84^ ^]^ These two ligands can undergo an acid–base reaction further leading to an increased complexity in ligand composition, which requires additional consideration for achieving ligand shell engineering.^[^ ^84^, ^85^, ^86^ ^]^ Other types of organic ligands and small inorganic molecules have been incorporated into modified synthetic procedures,^[^ ^87^, ^88^, ^89^, ^90^ ^]^ or included postsynthesis as additives to tune LHP NC properties.^[^ ^91^, ^92^, ^93^, ^94^ ^]^ Additives, such as silica, polymer, and molecular organic framework (MOF) precursors have also been considered for encapsulation purposes to further prepare these sensitive LHP NCs for applications.^[^ ^95^, ^96^, ^97^, ^98^ ^]^

In light of the increased complexities and rapid developments of the LHP NC systems and the important roles that the ligands play, understanding and engineering the surface ligand shell of LHP NCs is inarguably critical and inevitable for pushing the material to a wide practice of applications.^[^ ^99^ ^]^ While there are many excellent comprehensive reviews regarding LHP NCs in general,^[^ ^38^, ^39^, ^40^, ^41^, ^79^ ^]^ this review solely focuses on the importance and developments of surface ligands for LHP NCs. Specifically, we will cover the recent advances that have been made toward understanding and controlling the ligand shell in LHP NCs and the effects of such ligands on the materials’ quality and properties.

## 2 Ligand Shell Identification of LHP NCs

Organic ligands are utilized for a large majority of bottom‐up NC syntheses due to their ability to aid in precursor dissolution, to cap the surface of the resulting NCs in order to halt growth at the nanoscale, and to offer colloidal stability and prevent NC aggregation over long‐term storage. There are three types of ligands according to the covalent bond classification: L‐, X‐, or Z‐ type, which are classified depending on the number of electrons that the neutral ligand donates to the NC surface metal and nonmetal centers (2, 1, or 0, respectively).^[^ ^84^ ^]^ Without loss of generality, this section will focus on the characterization of the ligand shells for all inorganic CsPbBr_3_ LHP NCs.

### 2.1 Native Ligand Shell of As‐Synthesized CsPbBr_3_ NCs

**Figure 2:**
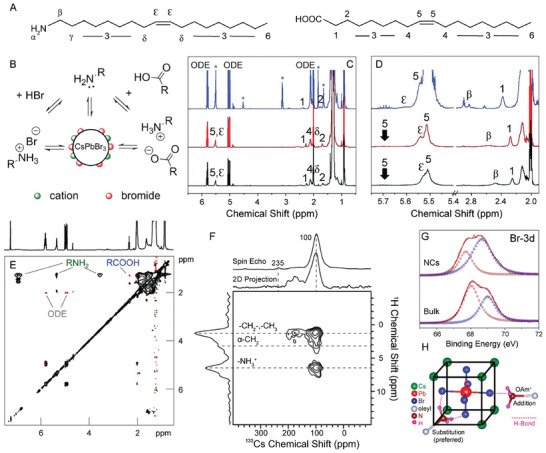
Ligand shell characterization. A) Molecular structure of oleylamine (OAm, left) and oleic acid (OA, right), the two most common ligands utilized in LHP NC synthesis. B) Schematic of the possible ligand–NC interactions in LHP NCs based on the precursors used in synthesis. A,B) Reproduced with permission.^[^ ^84^ ^]^ Copyright 2016, American Chemical Society. C) ^1^H NMR spectra of as‐synthesized CsPbCl_3_ (black), CsPbBr_3_ (red), and CsPbI_3_ (blue) NCs. Peak numbering corresponds to the hydrogen atom numbering shown in (A). D) Zoomed in regions of the ^1^H NMR spectra shown in (C). C,D) Adapted with permission.^[^ ^103^ ^]^ Copyright 2019, The Royal Society of Chemistry. E) NOESY spectrum of the ligand shell of CsPbBr_3_ NCs. Adapted with permission.^[^ ^84^ ^]^ Copyright 2016, American Chemical Society. F) Solid‐state ^133^Cs spin echo and 2D ^1^H→^133^Cs CP‐HETCOR NMR spectra. Adapted with permission.^[^ ^112^ ^]^ Copyright 2020, American Chemical Society. G) XPS spectra of the Br 3d peak for MAPbBr_3_ NCs compared to bulk. Red and blue dotted lines represent the fitting results of the spectra into two peaks. Adapted with permission.^[^ ^45^ ^]^ Copyright 2015, American Chemical Society. H) Schematic representation of possible interactions of oleylammonium with the LHP surface. Oleylammonium can directly add to the surface or can substitute into the “A” cation sites. Adapted with permission.^[^ ^113^ ^]^ Copyright 2017, American Chemical Society.

Most commonly, the following metathesis reaction is used between cesium oleate and lead bromide to form CsPbBr_3_ perovskite NCs

(1) $$2\mathrm{Cs}\left(\mathrm{OOCR}\right)+3\mathrm{PbB}r_{2}\;\to 2\;\mathrm{CsPbB}r_{3}+\mathrm{Pb}{\left(\mathrm{OOCR}\right)}_{2}$$

where OOCR is typically oleate (C_18_H_33_O_2_). In addition, oleic acid (OA) and oleylamine (OAm) are added to the reaction mixture as capping ligands (**Figure** **2**A). These two ligands can undergo an acid–base reaction to produce oleate and oleylammonium. As a result, possible species including lead oleate, oleylammonium bromide, OA, and OAm can be present in the as‐synthesized colloidal solution, all of which can potentially bind to the NC surface (Figure 2B).^[^ ^84^ ^]^ The organic components of these species can be detected using ^1^H nuclear magnetic resonance (NMR) spectroscopy, which has been heavily utilized to understand ligand binding at the NC surface.^[^ ^84^, ^100^, ^101^, ^102^, ^103^, ^104^ ^]^ In general, ligands associated with the NC surface appear broadened in the ^1^H NMR spectrum as a result of slower molecular tumbling effects, shorter T_2_ relaxation times, and/or NC inhomogeneity.^[^ ^103^, ^104^ ^]^ In the case of OA and OAm, peaks near the unsaturated C=C (*ε*,   *δ*,   5,   4) and near the functional groups (*α*, *β*,   *γ*,   1,   2) can be detected and identified individually as shown in Figure 2A,C–D.^[^ ^103^ ^]^ More specifically, peaks associated with hydrogen atoms adjacent to the molecules’ functional groups (*β*,   1  for OAm and OA, respectively) shift compared to controls, pointing toward a partial acid–base reaction (Figure 2A,C–D).^[^ ^103^ ^]^ Additionally, peaks for the OAm/oleylammonium species appear more broadened compared to the OA/oleate peaks, suggesting a stronger interaction between OAm and the NC surface (Figure 2C,D).^[^ ^103^ ^]^

Ligand shell coverage can be further elucidated using other 2D NMR techniques including nuclear overhauser effect spectroscopy (NOESY) and diffusion ordered spectroscopy (DOSY).^[^ ^104^, ^105^ ^]^ 2D NOESY is typically utilized to determine the average proximity between ^1^H nuclei within a sample.^[^ ^106^ ^]^ Specifically cross‐peaks will appear in a NOESY spectrum due to dipole–dipole interactions between ^1^H nuclear spins that are within ≈0.4  nm apart in space.^[^ ^106^ ^]^ For NC‐ligand interactions, surface‐bound ligands can be distinguished from free ligands due to their slow molecular tumbling, which greatly affects their NOE, and results in the build‐up of negative cross peaks (identical sign to the diagonal).^[^ ^107^ ^]^ Alternatively, free ligands within the colloidal solution tumble more quickly, and have positive cross peaks (opposite sign to the diagonal).^[^ ^107^ ^]^ For CsPbBr_3_ NCs synthesized with OA and OAm, the NOESY spectrum shows that residual 1‐octadecene (ODE, used as solvent in NC synthesis) and OA are not bound to the NC surface as a result of the positive, red cross peaks (Figure 2E, red cross peaks).^[^ ^84^ ^]^ On the other hand, the OAm species exhibited negative, black cross peaks, further confirming that the amine ligand is the major organic species interacting with the NC surface (Figure 2E, black cross peaks).^[^ ^84^ ^]^ Evidence of the acid–base reaction between OA and OAm coupled with the NOESY spectrum that reveals an amine species–NC interaction, strongly points toward an oleylammonium bromide surface passivation.^[^ ^84^ ^]^

The extent of oleylammonium binding can be further elucidated using DOSY. DOSY measurements, which gauge the diffusion coefficients of organic molecules in solution, can be linked to the size and shape of a NC core via the Debye–Einstein Equation (Equation (2))

(2) $$D\;=\frac{k_{B}T}{f}\;$$

where *D* is the diffusion coefficient, *k*_B_ is the Boltzmann constant, *T* is the temperature, and *f* is a friction factor dependent on NC morphology.^[^ ^84^, ^108^, ^109^ ^]^ If the measured species are considered spherical, the equation can be simplified, and the diffusion coefficient can be expressed using the Stokes–Einstein Equation (Equation (3))

(3) $$D\;=\frac{k_{B}T}{c\pi \eta r_{H}}\;$$

where *c* is a specific spherical shape‐dependent parameter, *η* is the solvent viscosity, and *r*_H_ is the hydrodynamic radius of the measured species.^[^ ^84^, ^108^, ^109^ ^]^ This equation allows for the direct calculation of the hydrodynamic radius of a spherical species using the diffusion coefficients measured in DOSY.^[^ ^108^, ^109^ ^]^ In general, smaller species are able to diffuse more quickly throughout the solution compared to larger ones.^[^ ^108^, ^109^ ^]^ As such, a free ligand will have a much faster diffusion compared to the same ligand strongly bound to a NC.^[^ ^109^ ^]^ Furthermore, DOSY measurements of ligands strongly bound to different sized NCs can be used for an accurate determination of the NC hydrodynamic size using Equation (3).^[^ ^109^ ^]^ This technique has been demonstrated for thiol‐capped gold nanoparticles and platinum‐dendrimer nanoparticle systems to determine nanoparticle sizes that matched well with transmission electron microscopy (TEM) measurements.^[^ ^109^, ^110^ ^]^

DOSY has also been utilized to determine the lability of the ligand shell in CsPbBr_3_ NCs. First, the cubic shape of the CsPbBr_3_ NCs was taken into account in Equation (3), and *c* was adjusted such that *c* = 0.6*6d*, where *d* is the average edge length of the nanocube.^[^ ^84^ ^]^ A diffusion coefficient of 166 ± 18 mm^2^ s^−1^ was measured for the oleylammonium species in the CsPbBr_3_ NC colloidal solution, which using Equation (3), resulted in a calculated nanocube edge length of 3.7  nm.^[^ ^84^ ^]^ While the measured diffusion coefficient was much slower than the control measurement of free oleylammonium bromide (361 mm^2^ s^−1^), the calculated edge length was smaller than the NC size measured using TEM (≈10  nm).^[^ ^84^ ^]^ This discrepancy in size indicates a highly labile ligand shell where the oleylammonium ligands are able to quickly exchange between bound and free states.^[^ ^84^ ^]^ This dynamic ligand state results in an intermediate diffusion coefficient which is a weighted average of the bound and free fractions of oleylammonium.^[^ ^84^, ^103^ ^]^ Using an average edge length of 10  nm, Equation (3) was applied to calculate an expected diffusion coefficient of 60 mm^2^ s^−1^ for oleylammonium ligands strongly bound to the NC surface.^[^ ^84^ ^]^ Then, the diffusion coefficients for the free ligands and the colloidal solution as well as the expected diffusion coefficient for strongly bound ligands were used to determine the percentage (*x*) of bound ligands in the CsPbBr_3_ NC sample using the following equation (Equation (4))

(4) $$x\;=\frac{D_{\mathrm{free}}-D_{\mathrm{meas}.}}{D_{\mathrm{free}}-D_{\mathrm{bound}}}\;$$

where *D*_free_ is the diffusion coefficient of free oleylammonium (361 mm^2^ s^−1^), *D*_meas._ is the measured diffusion coefficient of the colloidal solution (166  ± 18 m^2^ s^−1^), and *D*_bound_ is the calculated diffusion coefficient for oleylammonium ligands strongly bound to a NC with an edge length of 10  nm (60 mm^2^ s^−1^).^[^ ^84^ ^]^ Thus, using Equation (4), it was estimated that 65% of the oleylammonium ligands are bound to the NC surface, and undergo rapid exchange with the surrounding free ligands.^[^ ^84^ ^]^

### 2.2 Surface Termination of LHP NCs for Ligand Shell Composition

Following the determination of the major binding species in LHP NCs, it is vital to understand how oleylammonium is binding to the NC surface. However, due to many possible surface arrangements of the three‐component perovskite crystal, more in‐depth surface analysis may be required. When thinking more closely about the surface of LHP NCs, the nanocubes are terminated by neutral (100) crystal planes, which results in the possibility of cesium‐bromide or lead‐bromide NC surface terminations.^[^ ^111^ ^]^ A mixed cesium‐bromide and ligand terminated surface has been proved in solid‐state ^133^Cs and ^207^Pb  NMR experiments.^[^ ^112^ ^]^ The ^133^Cs spin echo NMR spectrum contained an additional peak at 235  ppm that was not present in the 2D ^1^H → ^133^Cs cross‐polarization heteronuclear correlation (CP‐HETCOR) spectrum and was attributed to surface Cs (Figure 2F).^[^ ^112^ ^]^ Conversely, both the ^207^Pb  spin echo and 2D ^1^H → ^207^Pb  CP‐HETCOR spectra only possessed signal from core Pb^2+^ ions, supporting a CsBr terminated surface.^[^ ^112^ ^]^ Presence of Br^–^ on the NC surface has been demonstrated experimentally through X‐ray photoelectron spectroscopy (XPS) measurements for both MAPbBr_3_ and CsPbBr_3_ NCs.^[^ ^45^, ^113^, ^114^ ^]^ In these measurements, two Br 3d peaks were observed due to the presence of both inner and surface ions for both bulk and nanosized particles (Figure 2G).^[^ ^45^ ^]^ Differences in the intensity ratio between the two peaks in the NCs compared to the bulk material indicated the presence of high concentrations of Br^–^ present on the NC surface.^[^ ^45^ ^]^

With oleylammonium being the major binding species for CsPbBr_3_ NCs, it has the ability to interact with the cesium‐bromide NC surface through the formation of hydrogen bonds, via either direct ligand addition, or substitution of the surface Cs^+^ cations.^[^ ^113^ ^]^ The substitution mechanism, however, results in the formation of a higher number hydrogen bonding interactions, which is more favored (Figure 2H).^[^ ^113^ ^]^ This substitution mechanism can be viewed either as organic cations occupying some of the surface “A” sites in the perovskite structure or as oleylammonium bromide surface passivation.

## 3 Ligand Roles in the Direct Synthesis of LHP NCs and their Effect on LHP NC Properties

While a variety of synthesis protocols and modifications have been reported over the past seven years, a typical synthesis of LHP NCs involves either a LARP or a hot injection procedure. Although additional synthetic techniques including top down methods such as ball milling^[^ ^115^, ^116^, ^117^ ^]^ and liquid‐phase exfoliation,^[^ ^118^ ^]^ and other bottom up methods including microwave,^[^ ^119^, ^120^ ^]^ ultrasonication,^[^ ^121^, ^122^ ^]^ solvothermal method,^[^ ^123^, ^124^ ^]^ and seeded growth^[^ ^125^ ^]^ have been reported, this review will mainly focus on the two aforementioned methods (i.e., LARP and hot injection methods). In this section descriptions of these two synthetic methods will be presented and the use of alkylamine and alkyl carboxylic acid ligands in these two syntheses will be explored. Specifically, the effect that these ligands have on optical properties, NC morphology and size, and superstructural assembly will be covered. Finally, ligands with varying functionalities and/or encapsulation materials that have been substituted into LARP and hot injection procedures will be introduced.

### 3.1 LARP Synthesis of Hybrid Organic Inorganic Perovskite (HOIP) NCs

Hybrid organic inorganic perovskite (HOIP) NCs were first reported in 2014 through a reprecipitation‐type synthesis that utilized a precursor solution containing a mixture of MABr, a medium‐to‐long (C8‐C18) chain alkylammonium bromide (octylammonium bromide or octadecylammonium bromide) and PbBr_2_ along with OA and ODE.^[^ ^37^ ^]^ Addition of this precursor solution to acetone initiated the nucleation of the first‐ever nanocrystalline‐sized LHP materials.^[^ ^37^ ^]^ Careful selection and addition of OA and the alkylammonium salt afforded the ability to optimize NC quality, demonstrating the importance of ligands in LHP NC synthesis in even the earliest works.^[^ ^37^ ^]^

**Figure 3:**
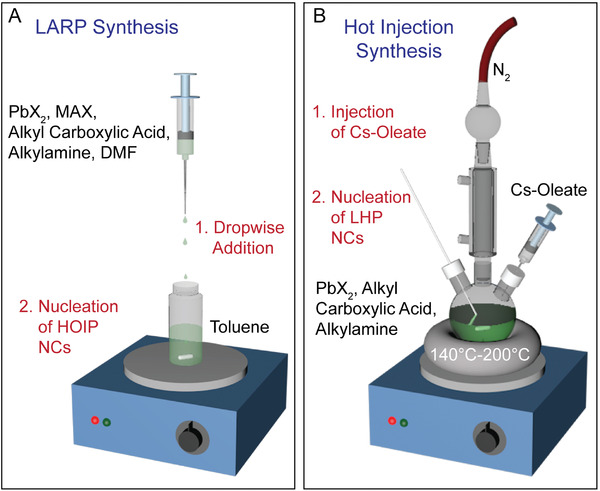
LHP NCs synthesis methods. A) Schematic representation of the LARP synthesis technique. B) Schematic representation of the hot injection synthesis technique.

Further improvement of this synthetic method led to the development of the LARP technique, which became a common method for synthesizing HOIP NCs (**Figure** **3**A) due to the volatile nature of small organic amines (i.e., MA^+^) utilized in the A site of the perovskite structure.^[^ ^45^ ^]^ In general, the synthesis involves the dissolution of precursors (commonly PbX_2_, MAX, and organic ligands) into what is termed a “good solvent” to form a precursor solution. This is followed by dropwise addition of the precursor solution into what is termed a “bad solvent” (Figure 3A). Good solvents most commonly include polar dimethylformamide (DMF) or dimethyl sulfoxide (DMSO), which, in the presence of ligands, possesses the ability to dissolve the perovskite precursors. On the other hand, the bad solvents that are heavily utilized include toluene, or chloroform, which are incapable of solubilizing the precursor salts. Upon the addition of the precursor solution to the bad solvent, sudden changes in polarity initiate NC nucleation which is followed by growth.^[^ ^45^ ^]^

The ligands utilized in this LARP procedure were shown to be vital in the crystallization process and overall stability of the NCs. A set of control reactions demonstrated that prolonged NC stability could only be realized in cases where both alkylamine and alkyl carboxylic acids were incorporated into the precursor solution during LARP synthesis.^[^ ^45^ ^]^ In looking more closely at the amine species, exclusion of *n*‐octylamine from the precursor solution resulted in NC aggregation, while increasing the alkyl chain length, like for the case of dodecylamine or hexadecylamine, could control the crystallization process and result in colloidal NCs.^[^ ^45^ ^]^ Long‐term stability, however, was compromised upon omission of OA.^[^ ^45^ ^]^ Taken together, the alkylamine ligand was identified to contribute to reaction kinetics, while the alkyl carboxylic acid prevents against particle aggregation and possesses a vital role in determining NC stability.^[^ ^45^, ^126^ ^]^

### 3.2 Hot Injection Technique for the Synthesis of All‐Inorganic CsPbX_3_ NCs

Developed alongside the LARP technique, the hot injection procedure has been specifically useful for synthesizing inorganic CsPbX_3_ NCs. Hot injection techniques are commonplace in the formation of conventional QD NC materials, and they rely on the quick injection of a precursor solution at elevated temperatures to spark NC nucleation.^[^ ^2^ ^]^ The injection is typically followed by rapid cooling in order to arrest further nucleation and promote the growth of monodispersed NCs.^[^ ^2^, ^12^ ^]^ This hot injection technique has been widely applied for the synthesis of all inorganic LHP NCs (Figure 3B).^[^ ^43^ ^]^ Hot injection has not been commonly applied for the synthesis of HOIP NCs (especially MAPbX_3_) because the required high temperatures often result in synthetic difficulties due to the volatility of the organic halide precursors. The first CsPbX_3_ NCs synthesized by hot injection were reported in 2015, where cesium oleate was injected into a hot ODE solution containing a lead halide salt precursor along with OA and OAm (Figure 3B).^[^ ^43^ ^]^ Focusing on the ligands themselves, typically a 1:1 mixture of OA to OAm was utilized in order to solubilize the precursor salts and stabilize the resulting NCs.^[^ ^43^ ^]^ In addition to this synthetic method, new hot injection procedures have been recently developed in order to decouple the halide and lead precursors.^[^ ^127^, ^128^ ^]^ For example, in a now widely utilized method, benzoyl halides or TMS‐X (halide precursors) are injected into a solution of lead acetate and cesium acetate with OA and OAm.^[^ ^58^, ^77^, ^127^ ^]^

Both the LARP and hot injection methods utilize long‐chain alkyl carboxylic acids and alkylamines to serve as ligands, where they work to assist precursor dissolution, control NC nucleation and growth, and stabilize the resultant NCs to prevent aggregation. Beyond the pioneering works, concentrations of these ligands have been altered to control NC properties including size, morphology, and optical performances. Additionally, a variety of other types of ligands have been explored as replacements for alkylamines and alkyl carboxylic acids for both the LARP and hot injection based syntheses, leading to fine‐tuned ligand conditions for improvement of optical and stability behaviors. The remainder of this section will review in more depth the role of alkyl carboxylic acids and alkylamines in both the LARP and hot injection syntheses, as well as introduce other types of ligands, encapsulation materials, and templates that have been incorporated into varying synthetic methods for LHP NCs.

### 3.3 Alkylamines and Alkyl Carboxylic Acids as Native Ligands

Both the LARP and hot injection perovskite NC syntheses typically rely on medium‐to‐long carbon chain (C8‐C18) alkylamines and alkyl carboxylic acids. This ligand combination, however, brings additional complications due to the acid–base reaction that can happen between the two species in nonpolar solvents.^[^ ^11^, ^85^, ^86^, ^129^ ^]^ Altering ligand compositions results in varying degrees of amine protonation, which can be probed using ^1^H NMR spectroscopy.^[^ ^85^, ^86^ ^]^ As discussed in Section  2, broadening and shifting of peaks associated with the amine species coupled with 2D NOESY spectra indicate that the typical ligand concentrations utilized in direct synthesis methods result in oleylammonium halide as the prominent binding species (Figure 2C,E).^[^ ^84^, ^103^ ^]^ As ligand compositions and identities are altered, it is imperative to understand the effect of those changes on ligand binding that occurs at the NC surface. In addition to changing the ligand ratios, simply aging precursor solutions has been shown to affect the molecular complexes present in solution, resulting in changes to LHP NC passivation and morphology.^[^ ^130^ ^]^ To eliminate some of the complications that arise from changes of OA and OAm ligand interactions as a result of the alteration of ligand ratio or through precursor aging, additives such as metal halide (e.g., ZnBr_2_, FeBr_3_, SnX_4_ (X=Cl, Br, I)), alkali metal, or alkaline earth metal salts, or small molecules like thiocyanate, have been incorporated into precursors during synthesis.^[^ ^111^, ^131^, ^132^, ^133^, ^134^, ^135^, ^136^ ^]^ Some of these additives were shown not only to further stabilize the NCs through inorganic passivation,^[^ ^111^, ^134^ ^]^ but also to trap the oleate species and promote a pure oleylammonium halide ligand shell.^[^ ^111^ ^]^ Other studies have focused on altering the concentrations and identities of the alkylamine and alkyl carboxylic acid ligands to understand the effect on NC optical properties, size, morphology, and self‐assembly and to target LHP NCs with ideal and controllable properties.^[^ ^137^, ^138^, ^139^ ^]^

#### 3.3.1 Effect on Optical Properties of LHP NCs

Addition of typical alkylamine and alkyl carboxylic acid ligands during LHP NC synthesis greatly helps to reduce the pathways toward nonradiative recombination, resulting in enhanced PL intensities.^[^ ^140^ ^]^ Specifically in the LARP synthesis, alteration of the ligand ratios helped elucidate the role that the ligands play both in stabilizing the NCs and boosting their optical performances.^[^ ^140^ ^]^ In terms of ligand selection, long‐chain OA and *n*‐octylamine resulted in good‐quality NCs with enhanced stability.^[^ ^45^ ^]^ MAPbBr_3_ NCs prepared using the LARP procedures without the addition of any ligands exhibited low PL QYs of 0.05%, due to the presence of a large number of surface traps.^[^ ^140^ ^]^ Addition of ligands to these weakly emitting NCs resulted in an increase of PL intensity for all types of ligands introduced (OA, OAm, oleylammonium bromide) except for OA by itself.^[^ ^140^ ^]^ This result suggests that OA does not interact with the NC surface without any amine species present (similar to CsPbBr_3_ NC counterparts (Section  2)) and that ligands greatly help reduce pathways toward nonradiative recombination originating from surface defects.^[^ ^140^ ^]^

**Figure 4:**
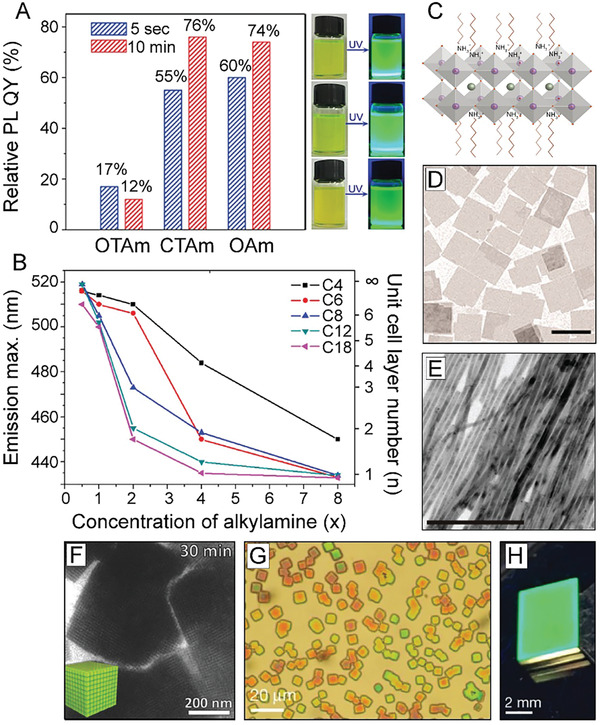
Effects of OA and OAm ligands in LHP NC growth and assembly. A) PL QY comparison of CsPbBr_3_ NCs synthesized with alkylamine ligands of varying length (left) and their corresponding optical pictures under visible and UV light (right). Adapted with permission.^[^ ^138^ ^]^ Copyright 2017, Elsevier. B) PL emission maxima as a function of concentration of added alkylamine in MAPbBr_3_ nanoplatelets synthesized with alkylamine ligands of varying carbon chain length. Increase of alkylamine resulted in a decrease of the number of unit cell layers in the LHP nanomaterials. Adapted with permission.^[^ ^165^ ^]^ Copyright 2016, American Chemical Society. C) Schematic illustration of 1‐unit cell layer thick MAPbX_3_ perovskite nanoplatelets. Protonated alkylamines reside in “A” position sites restricting further growth in that direction. Reproduced with permission.^[^ ^164^ ^]^ Copyright 2015, American Chemical Society. D) TEM image of CsPbBr_3_ nanosheets synthesized using a combination of short and long carbon chain alkylamines. (Scale bar is 1  µm). Reproduced with permission.^[^ ^160^ ^]^ Copyright 2016, American Chemical Society. E) TEM image of CsPbBr_3_ nanowires synthesized through altering the ratios between alkyl carboxylic acids to alkylamines. (Scale bar is 200  nm). Reproduced with permission.^[^ ^155^ ^]^ Copyright 2016, American Chemical Society. F) TEM image of CsPbBr_3_ supercrystals formed after 30 min synthesis time. Reproduced with permission.^[^ ^183^ ^]^ Copyright 2018, Wiley‐VCH. G) Optical microscopy image of a large‐area self‐assembly of LHP NCs achieved through solvent evaporation. H) Photograph of the green PL of an assembly of LHP NC superlattices under UV illumination. G,H) Reproduced with permission.^[^ ^184^ ^]^ Copyright 2018, Springer Nature.

Further studies altering the ligand concentrations and identities have also been performed to enhance the optical properties of inorganic LHP NCs, including their PL QYs and photostabilities.^[^ ^138^, ^141^, ^142^, ^143^, ^144^ ^]^ In one example, increasing the chain length of alkylamines used in a hot injection synthesis of CsPbBr_3_ NCs resulted in markedly improved PL QYs, demonstrating that carbon chain length has a strong correlation with the NCs’ optical properties (**Figure** **4**A).^[^ ^138^ ^]^ Moreover, direct use of oleylammonium bromide (OAmBr) was also found to alter the optical performances of the resulting NCs.^[^ ^141^, ^142^, ^143^ ^]^ In one case, OAmX was utilized as both a ligand and halide source, and injection into a Pb^2+^‐ and Cs^+^‐containing precursor solution initiated NC nucleation, resulting in CsPbBr_3_ NCs with near‐unity PL QYs.^[^ ^143^ ^]^ Use of this preformed salt helped to enhance crystalline stability at the high temperatures required in the NC synthesis.^[^ ^143^ ^]^ On the other hand, altering the chain length of the alkyl carboxylic acid was also shown to affect the PL QY of resultant CsPbX_3_ LHP NCs in a room temperature synthesis method.^[^ ^145^ ^]^ Specifically, increasing the alkyl chain length from butyric acid to oleic acid resulted in an increased PL QY, from 20–30% for butyric acid‐capped LHP NCs to over 80% for LHP NCs capped with either myristic or oleic acid.^[^ ^145^ ^]^

#### 3.3.2 Effect on Morphology and Size of LHP NCs

In addition to boosting the optical performances, changing the carbon chain lengths and ratios between the organic alkyl carboxylic acid and alkylamine ligands have been shown to yield LHP NCs with a variety of morphologies including nanospheres,^[^ ^146^, ^147^, ^148^ ^]^ nanocubes,^[^ ^146^, ^149^, ^150^, ^151^, ^152^ ^]^ nanorods,^[^ ^146^, ^147^ ^]^ nanobars,^[^ ^150^ ^]^ nanowires,^[^ ^149^, ^150^, ^153^, ^154^, ^155^ ^]^ nanoplatelets,^[^ ^146^, ^147^, ^148^, ^149^, ^150^, ^151^, ^152^, ^156^, ^157^, ^158^, ^159^ ^]^ and nanosheets^[^ ^147^, ^148^, ^160^ ^]^ using both modified LARP and hot injection synthesis methods. To explain the effects of precursor identity and ratio on the NC morphology, different mechanisms have been proposed including micellar transition mechanisms,^[^ ^146^ ^]^ seed‐mediated or ligand‐induced precursor activation,^[^ ^151^, ^161^, ^162^ ^]^ and surfactant‐directed 1D growth.^[^ ^153^ ^]^ Apart from these proposed mechanisms, the most heavily studied ligand–NC interaction is “A” site substitution with the protonated amine ligand as described above in Section  2 (Figure 2H).^[^ ^113^ ^]^ This “A” site substitution has been commonly exploited to synthesize lower‐dimensional nanoplates and nanosheets.^[^ ^163^, ^164^ ^]^ Specifically, increasing the concentration of alkylamine has been shown alter the thickness of LHP nanoplatelets down to as thin as a single unit cell layer.^[^ ^165^, ^166^, ^167^ ^]^ Blue shifts in absorption and PL emission features were observed due to the vertical dimension of the obtained nanoplatelets entering into the strong quantum confinement region (Figure 4B).^[^ ^165^, ^166^, ^167^ ^]^ In this proposed model for nanoplatelet growth, protonated alkylammonium cations can occupy “A” site positions in the perovskite structure and block growth along the [001] crystallographic direction, resulting in lower‐dimensional perovskite materials (Figure 4C).^[^ ^164^ ^]^ As such, identity and protonated proportion of the alkylamine can give control over resulting nanoplatelet thickness, which can be observed through optical, TEM, and atomic force microscopy (AFM) measurements (Figure 4D).^[^ ^137^, ^160^, ^168^, ^169^ ^]^ This shape‐directing role of alkylammonium has also been exploited to synthesize other unique perovskite structures including nanorods (Figure 4E), and HOIP core–shell structures.^[^ ^126^, ^170^ ^]^ In contradiction to this proposed growth model, MAPbBr_3_ nanomaterials synthesized using the LARP method with OA and OAm resulted in nanoplatelets at lower concentrations of OAm and nanospheres at higher concentrations of OAm.^[^ ^171^ ^]^ Initially, upon increase of OAm concentration, a blue shift in emission was observed, indicative of a decrease in nanoplatelet thickness and an increase in quantum confinement.^[^ ^171^ ^]^ This was followed by a redshift due to a morphology transition to nanospheres.^[^ ^171^ ^]^ In this case, it was argued that at excessively high concentrations of OAm, the platelet structure could no longer accommodate all of the ligands, preferring instead to break apart and form spherical‐shaped particles with newly exposed surfaces for ligation.^[^ ^171^, ^172^ ^]^

Recently, advanced control over LHP NC facet growth through nanocluster formation techniques has been explored.^[^ ^173^, ^174^, ^175^ ^]^ In this method, CsPbBr_3_ nanoclusters are first formed at room temperature and then serve as monomers in both hot injection and heat up method synthetic procedures.^[^ ^174^, ^175^, ^176^ ^]^ Using this technique, a multitude of NC morphologies (e.g., dot, wire, platelet, cube)^[^ ^174^ ^]^ including a variety of polyhedron with newly exposed facets (e.g., rhombic dodecahedron with stabilized {200}, {020}, and {112} crystal planes) have been reported.^[^ ^175^, ^177^ ^]^ Low concentrations of OA and OAm ligands were key to the formation of isotropic noncube polyhedral morphologies, which led to the halide deficient conditions required for the morphology control.^[^ ^175^ ^]^ Addition of an oleylamine‐hydrohalic acid salt to the polyhedron NCs led to arm growth and the formation of a complex hexapod structure.^[^ ^176^ ^]^ In another example of the formation of multifaceted polyhedron CsPbBr_3_ NCs, an *α*‐halo ketone was used as a halide source.^[^ ^177^ ^]^ In this report, a 12 faceted rhombic dodecahedron formed due to the in situ formation of a tertiary ammonium species upon reaction with the *α*‐halo ketone, showing a dependence of the polyhedron geometry on ligand concentrations (i.e., tertiary ammonium ions).^[^ ^177^ ^]^ Beyond synthesis, these facet‐variable polyhedron LHP NCs have already shown promise in self‐assembly^[^ ^178^ ^]^ and photocatalysis.^[^ ^179^ ^]^

Altering the ligand chain lengths or concentrations has also been demonstrated to affect the resulting NC size. The initial LARP synthesis of MAPbX_3_ NCs utilized a variety of alkylamine and alkyl carboxylic acid ligands, and it was determined that the amine species controls the kinetics of NC formation, which greatly affects the NC size.^[^ ^45^ ^]^ In another study, mixed (FA/MA)PbBr_3_ NCs synthesized with alkylamines of varying carbon chain lengths (C6–C16) resulted in a change in lateral size from 15.4 ± 3.9  nm to 6.4 ± 0.9  nm and a transition from NPLs to smaller and smaller nanocubes.^[^ ^180^ ^]^ Specifically, as the ligand carbon chain length was increased, the ligand became more hydrophobic and had enhanced miscibility with the nonpolar solvent, resulting in an earlier stage nucleation which, in turn, reduced the NC size.^[^ ^180^ ^]^ A study of ligand interactions during the formation of CsPbBr_3_ NCs using microfluidic screening and four different ligands (alkylamine and alkyl carboxylic acid pairs with eight carbons and different degrees of branching) determined that changes observed in the NC PL, size and morphology were a result of the ligand acid–base ratio and reaction temperature.^[^ ^181^ ^]^ Finally, alkylammonium bromide precursors have also been used as size controlling agents where an increase in concentration led to a decreased NC size due to the presence of a higher amount of ammonium capping agents.^[^ ^182^ ^]^

#### 3.3.3 Effect on Superstructural Assembly Behaviors of LHP NCs

Identity of the alkylamine and alkyl carboxylic acid has also been shown to control interactions between the NCs, resulting in higher‐ordered assemblies. For example, in terms of nanoplatelets, it has been hypothesized that the lamellar structures observed in TEM measurements insinuate that organic mesostructures (dependent on ligand concentrations and identities) are formed during synthesis and serve as growth‐directing templates.^[^ ^163^, ^167^ ^]^ CsPbBr_3_ supercrystals have also been achieved through self‐assembly during the direct synthesis process over prolonged reaction times, using OA and OAm as surfactants, in cases of high NC concentrations (Figure 4F).^[^ ^183^ ^]^ Specifically, van der Waals forces between the long chain ligands were identified to promote assembly into NC‐film formations.^[^ ^183^ ^]^ When small amounts of OA and OAm (1/1000 by volume compared to toluene) were added to a monodisperse sample of CsPbBr_3_ NCs, large‐area superlattices with domain sizes up to 15  µm were achieved.^[^ ^42^ ^]^ The resulting supercrystals could then be further assembled into larger areas.^[^ ^42^ ^]^ Large‐area supercrystals have also been formed through a careful drying process using as‐synthesized CsPbBr_3_ NCs with native OA and OAm ligands (Figure 4G,H) and exhibited superfluorescence.^[^ ^184^ ^]^

Self‐assembly of CsPbX_3_ NCs capped with OA and OAm or octylamine to achieve growth via oriented attachment has also been demonstrated to form larger perovskite nanomaterials including nanowires, large nanocubes, and pearl necklace assemblies.^[^ ^130^, ^185^ ^]^ For example, CsPbBr_3_ NCs (capped with OA and OAm) were transformed into ultrathin nanowires within 5 min, and large nanocubes within 10 min upon exposure to ethanol.^[^ ^130^ ^]^ In this case, the self‐assembly and growth process is attributed to the destabilization of surface ligands following exposure to the polar solvents.^[^ ^130^ ^]^ In another example, nanowire and pearl necklace assemblies were observed from MAPbBr_3_ nanospheres shortly following nucleation and without the addition of any external stimuli.^[^ ^185^ ^]^ The mesoscale growth of the nanospheres and assembly into wires was driven by dipole–dipole interactions between the spheres themselves, along with van der Waals interactions between the ligands (OA and octylamine).^[^ ^185^ ^]^

#### 3.3.4 Other Sterically Hindered Amine and Chiral Amine Ligand Effects

Beyond altering ligand chain lengths and ratios, the most basic reported change to the ligand identity during synthesis is the replacement of long‐chain alkylamines with more sterically hindered amines and nitrogen‐containing organic molecules. Branched quaternary alkylammonium halide ligands, including tetraoctylammonium halide and didodecyldimethylammonium halide (DDAX), are the most commonly reported OAm replacements, and have been shown to result in stronger ligand attachment and higher NC stability.^[^ ^186^, ^187^, ^188^, ^189^, ^190^ ^]^ For example, didodecyldimethylammonium bromide (DDAB) has been demonstrated to result in higher PL intensities over an extended temperature range (from 20 to 280 K) compared to Cs‐oleate ligands.^[^ ^191^ ^]^ In addition, passivation with DDAB resulted in monoexponential PL lifetime (PL LT) decay over a large temperature range, indicating a more effective ligand passivation.^[^ ^191^ ^]^ Secondary alkylamines have also been employed in synthesis to control NC morphology and promote the formation of pristine nanocubes, as the increase in steric bulk compared to OAm blocked platelet formation.^[^ ^192^ ^]^ One secondary alkylammonium salt, namely diphenylammonoium bromide was used to passivate CsPbBr_3_ NCs, and endowed enhanced conductivity due to the ligand benzene ring pi‐conjugations.^[^ ^193^ ^]^ Beyond the above discussed ligands, sterically hindered pyridine along with other aromatic ammonium cations and imidazolium cations have been demonstrated to enhance stability or control the morphology of HOIP NCs through blocking NC growth along certain crystallographic directions, resulting in nanowires and thickness‐controlled nanoplatelets.^[^ ^194^, ^195^, ^196^, ^197^ ^]^ In another example, bulky, sphere‐like adamantylamine ligands were used to replace OAm, and resulted in NCs with high photostabilities.^[^ ^198^ ^]^ These NCs were later successfully incorporated into perovskite films where they demonstrated higher stability and excellent electronic properties.^[^ ^139^ ^]^ Finally, 1,8‐octyldiamine bromide, with two terminal amine functionalities, and tripodal tris(2‐aminoethyl) ammonium bromide, with three functional amine groups, have been used to passivate CsPbBr_3_ NCs, resulting in an improvement in defect passivation, charge transport, and stability.^[^ ^199^, ^200^ ^]^

Apart from sterically hindered alkylamines, chiral amines have been utilized in the synthesis of LHP NCs to achieve high circularly polarized luminescence (applications in information storage, asymmetric catalysis, agriculture, and photoelectric devices), where differential emission is achieved upon illumination with left‐ or right‐circularly polarized light.^[^ ^201^, ^202^ ^]^ Specifically, circularly polarized luminescence can be achieved by inducing chirality in emitters, in this case, through the use of chiral ligands.^[^ ^201^ ^]^ In one example, a small portion of the OAm ligands were replaced with (*R*)‐2‐octylamine in the synthesis of FAPbBr_3_ NCs.^[^ ^201^ ^]^ The NCs were able to achieve circularly polarized luminescence with a *g*_lum_ = 6.8 × 10^−2^ (*g*_lum_ = $2\times \frac{\mathrm{IL}-\mathrm{IR}}{\mathrm{IL}+\mathrm{IR}}$, where IL and IR are the intensities of the left‐ and right‐handed circularly polarized luminescence, respectively).^[^ ^201^ ^]^

### 3.4 Varying Ligand Identity and Binding Modes in LHP NC Synthesis

The combination of long‐chain organic carboxylic acids and amines have been demonstrated to be valuable in the synthesis of LHP NCs, enabling control over NC optical properties, morphology, and assembly behavior. These ligands still have limitations, however, especially evident in the instability and fragility of the LHP NCs caused by their highly dynamic binding nature. As a result, synthetic methods have been adjusted in order to incorporate a wider array of ligands to provide strong ligand binding and thus higher stability of the LHP NCs.

#### 3.4.1 Phosphine Ligands in the Synthesis of LHP NCs

**Figure 5:**
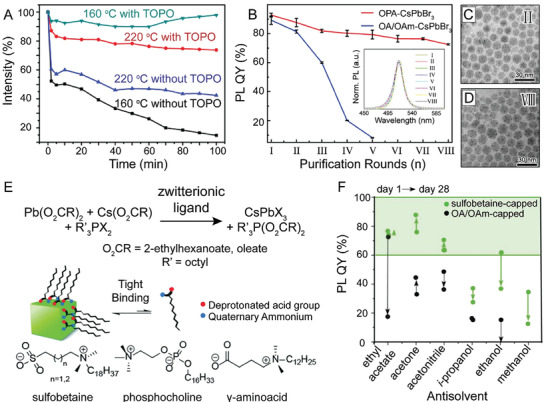
Varying ligand identity in the synthesis of LHP NCs. A) PL emission intensity as a function of ethanol treatment time for TOPO‐capped CsPbBr_3_ NCs (green and red) versus traditional OA/OAm‐capped CsPbBr_3_ NCs (blue and black) produced at varying synthesis temperatures. Adapted with permission.^[^ ^87^ ^]^ Copyright 2017, American Chemical Society. B) PL QY as a function of number of purification cycles with methyl acetate for octylphosphonic acid (OPA)‐capped CsPbBr_3_ (red) versus traditional OA/OAm‐capped CsPbBr_3_ NCs (blue). Inset shows lack of detectable change in PL emission of OPA‐capped CsPbBr_3_ NCs following multiple (8) rounds of purification. C,D) TEM images of the OPA‐capped CsPbBr_3_ NCs after 2 C) and 8 D) rounds of purification with methyl acetate. B–D) Reproduced with permission.^[^ ^207^ ^]^ Copyright 2018, American Chemical Society. E) Schematic image of the tight binding afforded by synthesizing CsPbX_3_ NCs with zwitterionic ligands. F) PL QY of NCs capped with 3‐(*N*,*N*‐dimethyloctadecylammonio)propanesulfonate (green) versus traditional OA/OAm (black) after two rounds of purification with varying solvents after 1 day and 28 days of storage time. E,F) Adapted with permission.^[^ ^88^ ^]^ Copyright 2018, American Chemical Society.

Ligands containing other types of functional groups have also been used in perovskite NC synthesis that utilize steric hindrance, strong NC binding, and the chelating effect to endorse higher NC stabilities. For example, highly branched trioctylphosphine oxide (TOPO) has been used along with traditional OA and OAm ligands during synthesis.^[^ ^87^ ^]^ The synthesized LHP NCs possessed a strong coordination to the P=O group confirmed by Fourier transform infrared (FTIR) spectroscopy measurements, which contained a characteristic C–P stretching peak at 1155 cm^–1^ even following NC purification.^[^ ^87^ ^]^ The TOPO‐capped LHP NCs possessed increased stability, demonstrated by their ability to maintain PL emission intensity after prolonged exposure to ethanol (**Figure** **5**A).^[^ ^87^ ^]^ Interestingly, in another report that utilized only TOPO and OA in the hot injection synthesis, ^1^H NMR measurements revealed that TOPO was not incorporated into the ligand shell, rather resulting in Cs‐oleate capping.^[^ ^203^ ^]^ The TOPO was instead found to form hydrogen bonding interactions with OA that could compete with the OA–PbBr_2_ interactions required for precursor dissolution.^[^ ^203^ ^]^ Thus, a delicate balance between OA and TOPO enabled control over reaction yield and NC size.^[^ ^203^ ^]^

Other phosphine ligands including trioctylphosphine (TOP), tributylphosphine, and diphenylphosphine have been shown to enhance NC stability as a result of the ligand steric effect.^[^ ^204^ ^]^ Furthermore, TOP is commonly added to reactions using traditional alkylamine and alkyl carboxylic acid ligands, where it has been shown to interact with PbI_2_ precursors to form reactive intermediates that accelerate nucleation and growth, leading to enhanced stability of the cubic perovskite *α* phase in CsPbI_3_ NCs.^[^ ^205^ ^]^ TOP has also been combined with lead stearate to form CsPbBr_3_ NCs where light, air, polar solvent and moisture stabilities were enhanced due to the proton‐free reaction environment.^[^ ^206^ ^]^

Alkylphosphonic acids, which contain two chelating sites giving the ligand strong interactions with the NC surface, have been used to replace OA and OAm. Unlike traditional OA/OAm capped NCs, LHP NCs capped with octylphosphonic acid could undergo multiple rounds of purification processes without losing particle integrity, facilitating their possible incorporation in devices (Figure 5B–D).^[^ ^207^ ^]^ In addition to the enhanced NC stability, other phosphonic acids, such as methyl, hexyl, octyl, tetradecyl, and octadecyl phosphonic acids, have been shown to lead to near unity PL QYs and almost single exponential PL LT decays in the resultant LHP NCs.^[^ ^208^ ^]^ The ^31^P NMR and TEM studies showed that the NCs were passivated by phosphonic acid anhydrides and hydrogen phosphonates, and possessed truncated octahedron morphology, respectively.^[^ ^208^ ^]^ In another example where a combination of diisooctylphosphonic acid, and TOPO was utilized, XPS measurements of the resulting NCs showed formation of Pb–O–P bonds which indicates that the P=O group of the phosphonic ligands interacts with the NC surface.^[^ ^209^ ^]^ Further FTIR characterization confirmed the interaction of TOPO with the NC surface and also revealed diisooctylphosphonic acid‐Pb^2+^ interaction due to shifting of the P=O stretching and P–OH stretching peaks, respectively.^[^ ^209^ ^]^ These interactions, however, were weak and the ligands could be removed through NC purification procedures.^[^ ^209^ ^]^ In another example, alkyl phosphonic acids have been coupled with TOP to produce more phase‐pure NCs with lower concentrations of impurities.^[^ ^210^ ^]^ Stability enhancement of the cubic perovskite *α* phase in CsPbI_3_ NCs has also been promoted by utilizing a combination of alkyl phosphoric acid, namely bis‐(2,2,4‐trimethyl pentyl)phosphoric acid, and OA.^[^ ^211^ ^]^

#### 3.4.2 Other Chelating and Zwitterionic Ligands in the Synthesis of LHP NCs

The use of dual‐functional and bidentate ligands has been shown to enhance LHP NC stability as a result of stronger ligand binding. For example, peptide molecules, like 12‐aminododecanoic acid, which contain both amine and carboxylic acid functional groups, afford synthetic control over size and optical properties through alteration of the sole, dual‐functional ligand.^[^ ^89^ ^]^ In another case, bidentate 2,2′‐iminodibenzoic acid was shown to strongly passivate the surface of CsPbI_3_ NCs, imparting additional NC stability and preventing the transition to the nonperovskite phase at room temperature.^[^ ^212^ ^]^ Succinic acid is a bidentate ligand that has been used in combination with OAm also leading to increased stability under ambient conditions.^[^ ^213^ ^]^ Similarly, L‐cystine, a trifunctional amino acid ligand, has been used to passivate MAPbBr_3_ NCs, resulting in highly stable assembled supercrystals due to electrostatic and van der Waals interactions between the NCs.^[^ ^214^ ^]^

Finally, it has been shown that zwitterionic ligands can promote tight ligand binding to perovskite NC surfaces resulting in enhanced ligand shell stability evidenced by prolonged optical stability compared to their OA/OAm‐capped counterparts (Figure 5E,F).^[^ ^88^ ^]^ In one example, natural soy lecithin was applied as a ligand in the synthesis of CsPbX_3_ NCs.^[^ ^215^ ^]^ This zwitterionic phospholipid not only served as an effective capping ligand, but also afforded high stability over a wide range of NC concentrations that is difficult to achieve with traditional alkylamine and alkyl carboxylic acid ligands. More specifically, the robust lecithin ligand shell maximizes NC–NC repulsion at high concentrations due to its brush‐like structure, thus preventing against aggregation.^[^ ^215^ ^]^

#### 3.4.3 Ligand‐Enabled Encapsulation of LHP NCs during Synthesis

**Figure 6:**
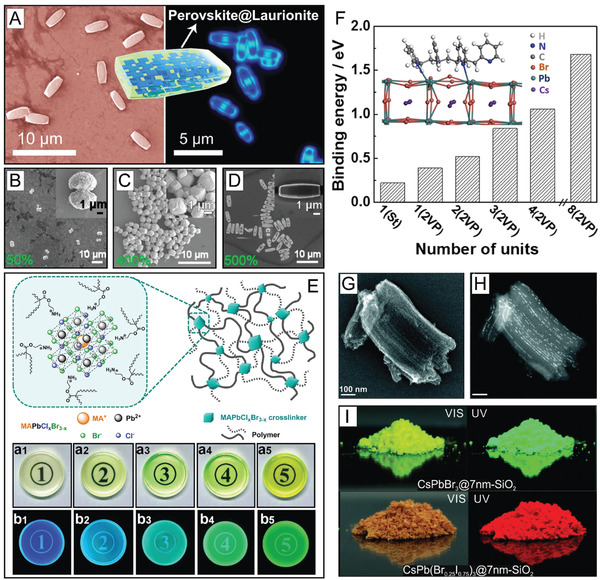
LHP NC encapsulation and templated growth synthetic strategies for enhanced NC stability. A) Laurionite‐type Pb(OH)Br encapsulation of reduced‐dimensional perovskite NCs. B–D) TEM images of perovskite@Pb(OH)Br core–shell microparticles formed using different volume ratios of OAm to OA (OA volume fixed to 75  µL). A–D) Reproduced with permission.^[^ ^216^ ^]^ Copyright 2020, American Chemical Society. E) Schematic illustration of MAPbCl*_x_*Br_3−_ *_x_* NCs ligated with polymerizable ligands and the resulting crosslinked polymer network (top) and transparent MAPbCl*_x_*Br_3−_ *_x_* NC crosslinked polymer network disks (diameter = 3  cm) under visible (above) and UV (below) illumination for NCs with increasing bromide compositions (decreasing *x*) (bottom). Reproduced with permission.^[^ ^217^ ^]^ Copyright 2018, American Chemical Society. F) Binding energy calculations for P2VP on an LHP NC surface with an increasing number of 2VP units. Inset shows a DFT simulation of a P2VP oligomer with four 2VP units on the LHP NC (100) surface. Adapted with permission.^[^ ^232^ ^]^ Copyright 2017, American Chemical Society. G) SE–STEM and H) HAADF–STEM images of CsPbI_3_ NCs in mesoporous silica matrices with a 7 nm pore size. I) Photographs of CsPbBr_3_ NCs (top) and CsPbI_3_ NCs bottom embedded in mesoporous silica with a 7  nm pore size under visible (left) and UV (right) light. G–I) Reproduced with permission.^[^ ^240^ ^]^ Copyright 2016, American Chemical Society.

Encapsulation strategies have been heavily employed in LHP NC systems to impart additional stability through protection of the LHP core from contact with air, water, polar solvents, etc. Ligands play an important role in the encapsulation process as they can enable growth of a shell material, enhance compatibility between the encapsulation material and the inorganic core, or undergo additional chemical processes such as crosslinking to directly encapsulate the LHP NCs.^[^ ^95^, ^216^, ^217^, ^218^ ^]^ In one example, OA and OAm were utilized to enable the growth of a laurionite‐type Pb(OH)Br material over LHP nanoplatelets to form LHP‐embedded microbricks through prolonging the LARP reaction time (**Figure** **6**A).^[^ ^216^ ^]^ This structure improved the environmental, thermal, chemical and optical stability, and additionally prevented postsynthetic ion exchange reactions between other LHP microbricks.^[^ ^216^ ^]^ In terms of the ligand role in this process, OAm was found to be needed for Pb(OH)Br formation, while OA had an effect on the resulting microparticle morphology (Figure 6B–D).^[^ ^216^ ^]^ Specifically, in increasing the ratio of OAm to OA a variety of morphologies, including microstars, microcylinders, dog‐bone shape microparticles, microdumbbells (Figure 6B), microrattles, microcubes, (Figure 6C) and microbricks (Figure 6D), could be formed.^[^ ^216^ ^]^ There have also been other examples of in situ formation of a protective shell barrier through altering precursor ratios to form inorganic perovskite‐analog shells. For example, shells of either CsPb_2_Br_5_ or Cs_4_PbBr_6_ have been grown onto inorganic LHP NCs, and have led to enhanced particle stability in water.^[^ ^219^, ^220^ ^]^ Alternatively, PbBr_2_ adlayers capped with DDAB have served as a shell material, demonstrating excellent stability against exposure to ethanol.^[^ ^221^ ^]^

Other, more complex ligands that serve a dual purpose in ligating the NC surface while forming protective shell coverage, have been heavily studied for enhanced NC stability. For example, (3‐aminopropyl) triethoxysilane (APTES) has been utilized as the sole ligand or in combination with OA and OAm, to directly form silica‐coated LHP NCs. Specifically, the amine functionality is able to interact with the NC surface, while the silyl groups can be hydrolyzed by trace water to form crosslinked silica shells.^[^ ^218^, ^222^, ^223^, ^224^, ^225^ ^]^ Polymer precursors can also produce more protective shells and enhance NC stability. In one example, poly(maleic acid anhydride‐*alt*‐1‐octadecene) was added to traditional OA and OAm ligands, where it was able to interdigitate into the normal capping ligands and be further polymerized with diamine for enhanced PL QYs and optical stability as a result of stronger ligand binding.^[^ ^95^ ^]^ Other polymerizable or crosslinkable precursors have been added to serve both as ligands and encapsulation materials during NC synthesis including polyimide, 4‐vinyl‐benzyl‐dimethyloctadecylammonium chloride, polystyrene (PS), poly(butyl methacrylate), poly(methyl methacrylate) (PMMA), polyethyleneimine, and lauryl methacrylate.^[^ ^226^, ^227^, ^228^, ^229^, ^230^, ^231^ ^]^ In general, these ligands lead to higher NC stabilities following crosslinking. For example, adding 2‐aminoethyl methacrylate hydrochloride as the sole ligand in a LARP synthesis yielded surface‐engineered crosslinkable MAPbCl*_x_*Br_3−_ *_x_* NCs (Figure 6E).^[^ ^217^ ^]^ Following synthesis, the methacrylic ammonium‐capped LHP NCs could undergo radical polymerization through the use of UV light and photoinitiator diphenyl(2,4,6‐trimethylbenzoyl)phosphine oxide to form rigid and transparent networks of LHP NC polymer composites (Figure 6E, bottom).^[^ ^217^ ^]^ These disks enhanced the long‐term stability, as the NCs were demonstrated to maintain 91% of their PL intensity following 30 days storage and 72% following immersion in water for 60 h.^[^ ^217^ ^]^

Diblock copolymer precursors can serve as nanoreactors and provide NC encapsulation.^[^ ^97^, ^232^, ^233^, ^234^, ^235^, ^236^, ^237^, ^238^ ^]^ They consist of tunable hydrophobic and hydrophilic blocks with different lengths, which are able to form reverse micelles where the NCs can form and become encapsulated. The hydrophobic groups then serve as robust protection barriers against UV light, O_2_, moisture, heat, water, and polar organic solvents.^[^ ^233^ ^]^ In one example, polystyrene‐*block*‐poly‐2‐vinylpyridine (PS‐*b*‐P2VP) diblock copolymer was added to toluene to form an inverse micelle (P2VP core and PS shell) which served both as a nanoreactor for LHP NC formation and as a protective ligand layer.^[^ ^232^ ^]^ During synthesis, PbBr_2_ precursors were first uptaken by the micelles, followed by the addition of CsBr, triggering nucleation.^[^ ^232^ ^]^ The P2VP block could coordinate with the PbBr_2_ precursor to enhance solubility, control LHP NC nucleation and growth, and cap the LHP NCs following nucleation while the hydrophobic PS block increased colloidal stability and protected the LHP NCs from moisture and polar solvents.^[^ ^232^ ^]^ Density functional theory (DFT) calculations were employed to gain further insight into the binding between the P2VP block of the inverse micelle and the LHP NC surface.^[^ ^232^ ^]^ With four simulated 2‐vinylpyridine (2VP) units on the (100) LHP NC surface, the 2VP units possessed an edge‐on conformation with all four N atoms facing the perovskite surface and two of the four forming coordinate bonds (Figure 6F, inset).^[^ ^232^ ^]^ Furthermore, the binding energy of the P2VP block was found to increase monotonically as the number of 2VP units increased (Figure 6F).^[^ ^232^ ^]^ This demonstrated that despite the steric hindrance of the bulky 2VP group, a considerable number of binding sites between the P2VP block and the LHP NC surface could form, enhancing NC stability.^[^ ^232^ ^]^

#### 3.4.4 Templated Growth of LHP NCs

In addition to altering ligand precursor identities, LHP NCs have been directly encapsulated into silica templates, polymers, MOFs and zeolites. Mesoporous silica can serve as a growth template where NC size, connectivity, and exposure to water can be controlled.^[^ ^239^, ^240^, ^241^ ^]^ In one example, commercially available mesoporous silica with varying pore widths was used to form both inorganic LHP and HOIP NCs through altering the drying temperature.^[^ ^240^ ^]^ In one sample using a template with a 7  nm pore width (Figure 6G), high angle annular dark field (HAADF) scanning‐TEM (STEM) was used to show the partial filling of the pores with CsPbI_3_ NCs (Figure 6H).^[^ ^240^ ^]^ Different LHP compositions could be achieved by altering the precursors used in synthesis, resulting in LHP NC impregnated mesoporous silica powders (Figure 6I).^[^ ^240^ ^]^ These powders showed high PL QYs (above 50%) due to the defect tolerant nature of LHP NCs, and improved processability, giving them potential for a wide range of applications.^[^ ^240^ ^]^ Kaolinite, a layered clay material made up of Al(OH)_3_ and SiO_4_ sheets, has been used as a matrix for the growth of CsPbX_3_ NCs, allowing for the initial deposition of Cs^+^, followed by treatment with PbX_2_ precursors.^[^ ^242^ ^]^ The initial Cs^+^ anchoring step prevents NC aggregation, resulting in composites with excellent stability and good processability.^[^ ^242^ ^]^

Polymers are able to undergo a swelling/deswelling process which can be taken advantage of in the formation of LHP NCs.^[^ ^243^ ^]^ Specifically, a variety of polymers including PS, acetonitrile butadiene styrene, cellulose acetate, polyvinyl chloride, polycarbonate, and PMMA are able to swell in DMF, a process during which LHP NC precursors can be incorporated into the polymer networks.^[^ ^243^, ^244^ ^]^ When the polymers are deswelled, solvent evaporation triggers NC nucleation.^[^ ^243^, ^244^ ^]^ Performing this encapsulation method with PS, polycarbonate, polyvinyl chloride, and acetonitrile butadiene styrene resulted in LHP NCs that were stable in water for two months with less than a 7% loss in PL QY.^[^ ^243^ ^]^ LHP NCs can also be encapsulated in polymers through control of crystallization processes. For example, polyvinylidene fluoride has been used to encapsulate LHP NCs, where the success of the composite formation was achieved through initial crystallization of the polymer film followed by NC nucleation.^[^ ^245^ ^]^

LHP NCs have also been grown in the pores of MOF films.^[^ ^98^, ^246^, ^247^ ^]^ In one case, a MOF film with nanosized pores (Cu_3_(1,3,5‐benzene tricarboxylate)_2_) was submerged in a PbI_2_ solution followed by introduction of MA^+^ solution.^[^ ^98^ ^]^ The synthesized NCs were monodisperse with sizes close to the MOF pore size.^[^ ^98^ ^]^ Similarly, MAPbI_3_ NCs have been formed in the pores of Fe‐porphyrin MOFs which could then serve as photocatalysts for CO_2_ reduction, as the catalytic Fe center of the MOFs were positioned close to the photocatalytic NCs.^[^ ^246^ ^]^ Zeolite‐Y has been used to form CsPbX_3_ NCs through an initial ion exchange to replace Na^+^ in the zeolite structure with Cs^+^, followed by introduction of PbX_2_.^[^ ^248^ ^]^ This stepwise synthesis procedure allowed for the formation of the LHP NCs in the zeolite pores without having to balance diffusion control for all three ion precursors.^[^ ^248^ ^]^ Finally, LHP NCs have also been nucleated on the surface of microcrystalline carriers which could be later shelled with NaBr, affording additional protection for enhanced stability.^[^ ^249^ ^]^

## 4 Postsynthetic Ligand Modifications in LHP NCs

LHP NCs have been demonstrated to have a highly labile ligand shell. This imparts additional benefits and/or complications to postsynthetic ligand treatments of LHP NCs, which are typically shown to have a great effect on the ligand shell composition and the resulting NC stability and properties. For example, even simple washing procedures of the as‐synthesized NCs have been reported to greatly affect the resulting ligand composition.^[^ ^250^ ^]^ Specifically, ligands can be removed with each washing step, causing a shift in ligand–NC equilibria and decreased stability resulting in optical performance loss.^[^ ^250^ ^]^ In one report, the use of acetone as a nonsolvent opposed to ethanol in CsPbX_3_ NCs with mixed Br/I composition resulted in a selective NC surface etching process to yield a bromide‐rich surface and higher NC stability.^[^ ^251^ ^]^ In other studies, ligand additives (typically alkylamines and alkyl carboxylic acids) have been added in‐between washing steps to maintain optical performance and/or to enhance photostability upon multiple rounds of purification.^[^ ^252^, ^253^ ^]^ Besides, even simpler treatments to LHP NC colloidal solutions, like dispersion processes, have been prone to greatly alter NC morphologies.^[^ ^254^, ^255^, ^256^ ^]^ As a result of the high sensitivity of the perovskite NC ligand shell composition on any postsynthetic treatment, care must be taken in order to maintain NC stability and/or to meet application‐driven requirements.^[^ ^29^, ^257^, ^258^, ^259^ ^]^

### 4.1 Ligands for Passivating LHP NC Surface Defects

LHP NCs initially emerged as promising materials due to their defect‐tolerant nature, a property that enabled the direct synthesis of highly emissive NCs, without the need for additional shell growth steps commonly utilized in traditional QD syntheses.^[^ ^12^, ^13^, ^43^, ^260^, ^261^, ^262^, ^263^, ^264^ ^]^ This defect tolerance, however, does not warrant the complete avoidance of some surface‐related defects. Specifically, the direct synthesis of LHP NCs typically utilizes lead‐rich conditions.^[^ ^43^, ^45^ ^]^ These lead‐rich conditions, combined with the labile nature of native OA and OAm ligands, inevitably leads to surface defects such as undercoordinated surface lead species and bromide vacancies.^[^ ^79^, ^131^, ^265^ ^]^ Therefore, additional postsynthetic ligand and additive passivation or heterostructure formation can be used to passivate some of the surface defects and boost the overall optical performances and/or NC stabilities.^[^ ^93^, ^94^, ^259^, ^260^, ^261^, ^262^, ^263^, ^264^, ^265^, ^266^, ^267^, ^268^ ^]^

#### 4.1.1 Intercalation of Small Additives and Addition of Excess Ligands for LHP NC Enhanced Surface Coverage

**Figure 7:**
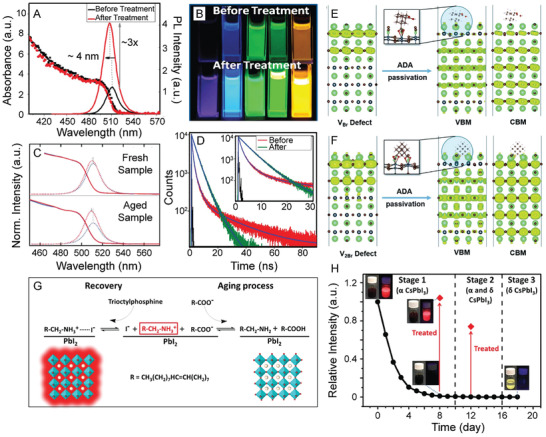
Enhancement of NC properties through postsynthetic surface treatments. A) Absorption and emission of CsPbBr_3_ NCs before (black) and after (red) a surface treatment with a tetrafluoroborate salt showing ≈3*x* PL emission intensity enhancement. B) Photographs of CsPbX_3_ NCs under UV illumination before (above) and after (below) surface treatment with a tetrafluoroborate salt showing brighter NC emissions following treatment. A,B) Reproduced with permission.^[^ ^93^ ^]^ Copyright 2018, American Chemical Society. C) Absorption and emission of untreated CsPbBr_3_ NCs (blue) and CsPbBr_3_ NCs that were treated with a thiocyanate salt (red) directly following synthesis (above) or after NC aging (below). Adapted with permission.^[^ ^94^ ^]^ Copyright 2017, American Chemical Society. D) PL LT spectra of CsPbBr_3_ NCs before (red) and after (green) tetrafluoroborate treatment showing transition toward monoexponential decay behavior. Adapted with permission.^[^ ^93^ ^]^ Copyright 2018, American Chemical Society. E,F) Charge density calculations using PBE0+SOC for CsPbBr_3_ with E) a single surface Br^–^ vacancy or F) two surface Br^–^ vacancies without (left) and with (right) 1,3‐adamantanedicarboxylic acid (ADA) passivation. Reproduced with permission.^[^ ^266^ ^]^ Copyright 2019, Royal Society of Chemistry. G) Schematic illustration of the loss of PL intensity in CsPbI_3_ NCs due to the acid–base reaction between OA and OAm (right) and PL recovery upon introduction of TOP (left). H) PL intensity of CsPbI_3_ NCs versus time (black) and change in PL intensity following TOP treatment performed at differing stages of the aging process (red). G,H) Reproduced with permission.^[^ ^275^ ^]^ Copyright 2020, American Chemical Society.

One successful method used to combat surface defects that arise as a result of lead‐rich surface environments is to postsynthetically incorporate additives which can passivate the undercoordinated Pb^2+^ ions. This technique has been heavily explored in LHP NCs, through the addition of a variety of different small organic molecules or inorganic complexes to the colloidal solution. In more detail, postsynthetic thiocyanate or tetrafluoroborate treatments have been shown to enhance the PL QY by repairing the lead‐rich surface (**Figure** **7**A,B).^[^ ^93^, ^94^ ^]^ This enhanced PL emission can also be achieved using the thiocyanate treatment in NC samples that have already aged, raising the PL QY to similar values as fresh NCs that undergo the same thiocyanate treatment (Figure 7C).^[^ ^94^ ^]^ The small molecule additives are able to access a limited amount of surface sites, and bind to and remove excess lead ions, resulting in more stoichiometric NC compositions and decreased surface traps.^[^ ^93^, ^94^ ^]^ This leads to a decrease in nonradiative recombination pathways, which can be validated through the (close to) monoexponential PL LT decay behavior of LHP NCs following the treatment (Figure 7D).^[^ ^93^, ^94^ ^]^ Thiocyanate treatment has also been demonstrated to yield other improvements to the LHP NCs including the suppression of trion generation, leading toward a decreased photocharging of the NCs during their use in devices,^[^ ^269^ ^]^ and suppression of NC flickering.^[^ ^270^ ^]^

Decreasing undercoordinated surface lead defects in LHP nanoplatelets can also be achieved by adding excess PbBr_2_ and ligands to the NCs postsynthetically.^[^ ^271^ ^]^ This treatment works in a somewhat opposite way to the other small molecule additives, adding in Pb^2+^ to fill in Pb^2+^ and Br^–^ surface vacancy sites while providing excess ligands to passivate undercoordinated surface ions.^[^ ^271^ ^]^ In another study, all of the LHP NC precursors (CsX and PbX_2_) were added to CsPbI_3_ NCs to improve PL stability over time.^[^ ^272^ ^]^ Addition of other metal chlorides including VCl_3_, SnCl_2_, NiCl_2_, PbCl_2_, SbCl_3_, BiCl_3_, ZnCl_2_, and CuCl, or organo‐chlorides such as RNH_3_Cl, have been used to maintain emission intensity and prevent transformation to the tetragonal phase in CsPbCl_3_ NCs during purification through the presence of surplus Cl^–^ ions.^[^ ^273^ ^]^ In another example, 1,3‐adamantanedicarboxylic acid (ADA) was added along with ZnBr_2_ to a colloidal solution of CsPbBr_3_ NCs to enhance the PL QY.^[^ ^266^ ^]^ Specifically, this dual passivation strategy renders the PL QY close to unity (97.1%) and facilitates enhanced stability (only 7% decrease in PL QY following 65 days storage).^[^ ^266^ ^]^ The ADA ligand is able to passivate Br^–^ vacancies which lead to trap states in the bandgap.^[^ ^266^ ^]^ DFT calculations showed that ADA was able to passivate one (Figure 7E) or two (Figure 7F) Br^–^ vacancies, resulting in a fully delocalized valence band maximum (VBM) and conduction band minimum (CBM) with trap‐free bandgaps.^[^ ^266^ ^]^ This indicated that the ADA ligand can shift the energies of Br^–^ vacancy defects to outside of the bandgap.^[^ ^266^ ^]^ On the other hand, similar to the PbBr_2_ treatment discussed above,^[^ ^271^ ^]^ ZnBr_2_ is able to fill in vacant Pb–Br sites on the LHP NC surface, eliminating a trap state located on the VBM.^[^ ^266^ ^]^ In total, through treatment with ADA and ZnBr_2_, both Br^–^ and Pb^2+^ vacancy defects can be passivated, suppressing nonradiative recombination and resulting in an enhanced PL.^[^ ^266^ ^]^

Contrary to the other examples of small molecule additives, NC surface treatment with nitrate ions works through peeling away cation defects on the NC surface, leading toward NCs with high PL QYs (≈85%).^[^ ^274^ ^]^ Similarly, oxalic acid has been added to mixed halide LHP NC colloidal solutions, where it is able to chelate to the NC surface, not only to remove excess Pb^2+^ or Cs^+^ ions which cause surface defects, but to also control halide compositions through the stronger binding of oxalic acid to chloride in comparison to bromide.^[^ ^267^ ^]^

Some ligands used in the direct synthesis of LHP NCs have been demonstrated to remove surface defects and boost PL QYs when added to the colloidal solution postsynthetically. For example, TOP was added to aged colloidal solutions of CsPbI_3_ NCs to regain losses in PL QYs and prevent the transition to the nonperovskite phase (*δ*‐phase) typically observed upon long‐term storage.^[^ ^275^ ^]^ While the TOP was shown to lack direct interaction with the NC surface, it was found to alter the equilibrium between OAm and olelyammonium to favor higher concentrations of oleylammonium and yield better passivation and improved NC properties (Figure 7G).^[^ ^275^ ^]^ Specifically, when TOP was added to aged CsPbI_3_ NCs prior to *δ*‐phase conversion, the weakened PL intensity could be recovered (Figure 7H).^[^ ^275^ ^]^ In another example, introduction of 1‐dodecanethiol was demonstrated to enhance the PL QY of OA and OAm‐capped CsPbBr_3_ NCs from 76.1% to 99.8%.^[^ ^276^ ^]^ 1‐dodecanethiol, as a softer X‐type Lewis base (as compared to OAm), is able to more efficiently passivate the undercoordinated Pb^2+^ species, thus boosting the optical performance.^[^ ^276^ ^]^

#### 4.1.2 Ligand Effect on Inorganic Heterostructure Formation on LHP NCs

Another effective strategy that has been commonly used for passivating surface defects and enhancing PL QY in traditional QDs (e.g., CdSe, InP QDs) is the growth of an inorganic shell around the NC core.^[^ ^12^, ^13^, ^261^, ^262^, ^263^, ^264^ ^]^ Although this strategy is not as successful in the case of LHP NCs, there have been reports of the growth of a PbS shell on CsPbI_3_ NCs, which gave the NCs enhanced stabilities in both particle integrities and PL QYs.^[^ ^277^ ^]^ 10  nm thick AlO*_x_* shells have also been grown around CsPbX_3_ NCs through an atomic layer deposition strategy, which can slow down anion exchange reactions.^[^ ^278^, ^279^ ^]^ On the other hand, adding a PbSO_4_ precursor to cap the NC core has been demonstrated to entirely halt ion exchange, making it possible to design devices with tandem perovskite layers while avoiding composition homogenization.^[^ ^268^ ^]^ A reverse core–shell structure with the growth of a HOIP shell layer around PbS QDs has also been demonstrated.^[^ ^280^ ^]^ The HOIP shell enabled the PbS core to retain its emission, while the core–shell structure remained intact over months of storage.^[^ ^280^ ^]^

Beyond core–shell structures, other heterostructures have also been achieved, leading to enhanced properties. In one example, utilizing the ligands present in the colloidal solution to reduce gold precursors (e.g., AuBr_3_), small Au NC islands were grown on the corners of LHP NCs, which have the potential to enhance the core LHP NC heterostructure's photocatalytic abilities.^[^ ^281^ ^]^ Improvement upon this Au‐deposition method showed that addition of PbBr_2_ to the colloidal solution along with AuBr_3_ prevented any exchange between Pb^2+^ in the perovskite structure with Au^+^ or Au^3+^ that could lead to the decomposition of the CsPbBr_3_ lattice.^[^ ^282^ ^]^ This alteration also promoted sized‐controlled Au island growth, dependent on the concentration of AuBr_3_ added to the colloidal solution.^[^ ^282^ ^]^ Similar to the Au–LHP NC heterostructures, CsPbX_3_–PbS heterostructures have been formed through the in situ growth of PbS onto the LHP NCs through the addition of hexamethyldisilathiane as a sulfur source.^[^ ^283^ ^]^ The resulting heterostructure possessed dual PL emission from both the CsPbX_3_ and PbS NCs, which could be further tuned through compositional changes of the CsPbX_3_ NCs or through adjusting the PbS NC size.^[^ ^283^ ^]^

### 4.2 Ligand Exchange and Encapsulation Strategies for LHP NCs

As mentioned above, stability is one of the major issues that has been plaguing LHP NCs every step of the way. Although these NCs are easily formed, allowing access to precise compositional and property tuning, their facile formation also yields an ease of destruction. Specifically, LHP NCs are sensitive to a variety of things, such as water, oxygen and light, present at ambient conditions. As a result, many postsynthetic ligand engineering strategies have been designed to tackle some of these stability issues. In addition, ligand exchange is one strategy that has been demonstrated to improve electrical conductivities of LHP NC‐based films, making the LHP NCs more ideal for their device applications. Furthermore, encapsulation of the LHP NCs and use of more robust coatings have also shown high potential for maintaining NC stability and integrity over long periods of storage time and use.

#### 4.2.1 Ligand Exchange Reactions for LHP NCs

Ligand exchange is a common strategy utilized in many NC systems in order to improve various NC properties and enable applications. Oftentimes, ligand exchange is exploited to adjust dispersibility in varying solvents, prepare for NC use in different applications, or to achieve ligand functionality that could not be attained during the direct synthetic process. Furthermore, ligand exchange has the potential to become one of the new frontiers of LHP NC research as a result of the high ligand lability, opening the door for a huge potential in surface manipulation.^[^ ^284^ ^]^ For example, ligand exchange has been demonstrated to occur as a result of simple purification procedures.^[^ ^284^ ^]^ Specifically, acetic acid formed via hydrolysis of a methyl acetate antisolvent used in purification was found to protonate surface‐bound oleate ligands, resulting in acetate‐passivated NCs.^[^ ^284^ ^]^

Although not heavily explored and exploited thus far, there are a few examples of ligand exchange reactions in LHP NCs in order to achieve improved NC‐film conductivity and enhanced optical properties.^[^ ^92^, ^285^, ^286^, ^287^, ^288^ ^]^ In one example, native ligands were replaced by X‐type thiol ligands in order to improve NC conductivity.^[^ ^92^ ^]^ Since thiols bind with exposed Pb^2+^ species present on the NC surface, a pre‐surface treatment where excess Pb^2+^ ions were added to the NC surface was completed prior to ligand exchange.^[^ ^92^ ^]^ This pretreatment provided more ligand binding sites on the surface, and thus improved final NC stability.^[^ ^92^ ^]^ Increased charge transport property has also been achieved through removal of long native organic ligands upon the addition of thionyl halide.^[^ ^285^ ^]^ In this solution‐phase ligand exchange, thionyl halide was able to react with the native ligands, leaving behind halide anions while retaining NC optical properties.^[^ ^285^ ^]^

Ligand exchange has also been completed utilizing amine or ammonium precursors. For instance, amines with varying carbon chain length (C4‐C8) have been successfully exchanged with OAm.^[^ ^289^ ^]^ Other quaternary alkylammonium halide salts, including DDAB/didodecyldimethylammonium chloride (DDAC), dodecyl‐dimethylammonium bromide, dimethyldodecylammonium bromide, and tetraoctylammonium bromide were also applied in ligand exchange reactions.^[^ ^286^, ^287^, ^288^ ^]^ These ligands have been shown to reconstruct the NC surface, therefore reducing the number of surface trap defects and enhancing the PL QY.^[^ ^286^, ^287^, ^288^ ^]^ DDAB, specifically, has been extensively utilized to enhance NC conductivities, making it a go‐to ligand for LHP NC device fabrications.^[^ ^286^, ^288^ ^]^ In another example, CsPbBr_3_ NCs were directly synthesized with short chain propionic acid and butylamine, followed by ligand exchange through adding different alkylamines and alkyl carboxylic acids during purification steps.^[^ ^290^ ^]^ Although ligands with shorter carbon chain lengths were tested, LHP NCs where short carbon chain ligands were replaced with OA and OAm were found to have the best device performances when incorporated into light emitting diodes (LEDs) due to their ability to form uniform, smoothly covered films.^[^ ^290^ ^]^

Possibly due to restrictions in direct synthesis, a number of exotic ligands were attached to the surface of LHP NCs via ligand exchange reactions. In terms of ligand‐introduced and ‐enhanced functionalities, octylphosphonic acid was employed in a ligand exchange reaction in CsPbBr_3_ NCs.^[^ ^91^ ^]^ The ability of forming Hydrogen bonds with neighboring ligands led to dramatically enhanced stabilities for NC morphology, crystal structure, and PL.^[^ ^91^ ^]^ Native ligands (i.e., oleate) have also been replaced with cinnamate acid for CsPbBr_3_ NCs which yielded an increased photoredox activity and dispersibility of the LHP NCs in polar solvents such as methyl acetate and dichloromethane.^[^ ^291^ ^]^ Fluorinated perfluorodecanoic acid has been used to replace native oleate and tetraoctylammonium ligands, resulting in an enhanced PL intensity (increased in PL QY from 72% to 90%), and a better stability against ethanol.^[^ ^292^ ^]^ Bidentate 2,2′‐iminodibenzoic acid replaced oleic acid through ligand exchange, resulting in a significant increase in PL QY, stability, and NC shelf life.^[^ ^293^ ^]^ Improvement of these properties was attributed to the two binding sites of the 2,2′‐iminodibenzoic acid ligand, leading to enhanced NC passivation.^[^ ^293^ ^]^ A polyzwitterionic ligand with even more NC binding sites (4–8 sulfobetaine zwitterionic groups per polymer) was designed with both alkyl chains and zwitterion groups laterally arrayed along the backbone of the polymer.^[^ ^294^ ^]^ CsPbBr_3_ NCs passivated with these multidentate polymer ligands through ligand exchange exhibited enhanced stabilities, with the ability to be kept in polar solvents for extended periods of time (2–8 months in ethanol).^[^ ^294^ ^]^

Finally, ligand exchange with more complex ligands has been utilized to achieve properties beyond NC stability. For example, perylene diimide ligand dyes have been used to replace native OA and OAm, resulting in Förster resonance energy transfer (FRET) from the NCs to the ligand dye.^[^ ^295^ ^]^ This opened the door to the possibility of new hybrid emissive systems with the potential to down‐convert high energy light emission.^[^ ^295^ ^]^ In another example, bifunctional *α*‐amino butyric acid was used to replace OA and OAm on CsPbBr_3_ NCs, and resulted in the first example of ligand exchange‐induced bandgap tuning.^[^ ^296^ ^]^ Specifically, the NCs were found to decrease in size during the ligand exchange process, resulting in a higher intensity and blue‐shifted PL.^[^ ^296^ ^]^ In another example, CsPbX_3_ nanocubes were transformed into nanoplates through a ligand‐mediated anion exchange reaction.^[^ ^297^ ^]^ In this reaction, dodecanethiol and AlX_3_ were added to the LHP nanocubes to simultaneously trigger an anion and ligand exchange, further enhancing the PL intensity over 74 times.^[^ ^297^ ^]^

#### 4.2.2 Postsynthetic Encapsulation Strategies in LHP NCs

**Figure 8:**
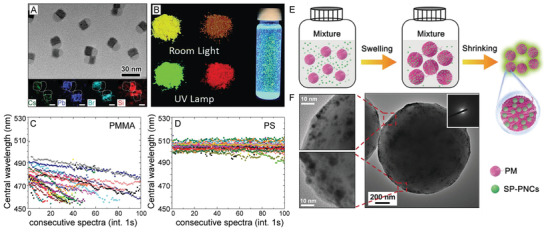
Postsynthetic encapsulation of LHP NCs. A) TEM image of the CsPbBr_3_/SiO_2_ Janus NCs (top) and elemental mapping results (bottom, scale bars = 10  nm). Reproduced with permission.^[^ ^302^ ^]^ Copyright 2018, American Chemical Society. B) Photographs of POSS‐coated NC powders (left) and a POSS‐NC suspension in water after 10 weeks storage under UV light (right). Reproduced under the terms of a Creative CommonsAttribution 3.0 International License.^[^ ^305^ ^]^ Copyright 2016, Royal Society of Chemistry. C,D) Central emission wavelength for different individual NCs embedded in C) PMMA and D) PS over time. Adapted with permission.^[^ ^314^ ^]^ Copyright 2019, American Chemical Society. E) Schematic image of the swelling/deswelling process utilized for incorporating LHP NCs into PS microspheres. F) TEM images of the LHP NCs incorporated into polymer spheres. Reproduced with permission.^[^ ^311^ ^]^ Copyright 2019, Wiley‐VCH.

Apart from ligand exchange reactions, creation of a stronger barrier between the solvent and the surface through partial or full encapsulation postsynthesis is another effective strategy to improve particle stability. Among many encapsulation materials, silica is arguably the most common and widely practiced in a variety of NC systems.^[^ ^298^, ^299^, ^300^ ^]^ Successful silica growth has been demonstrated on LHP NCs through the addition of tetramethyl orthosilicate (TMOS), which can hydrolyze quickly, which is a necessity for LHP NCs during the silica growth process.^[^ ^301^, ^302^, ^303^, ^304^ ^]^ As an example of partial silica coating, Janus type silica‐LHP NC hybrid structures could be formed through a sol‐gel process (**Figure** **8**A).^[^ ^302^ ^]^ These CsPbX_3_–SiO_2_ Janus particles were formed by mixing 0D Cs_4_PbBr_6_ NCs with TMOS in nonpolar hexane and then rapidly adding H_2_O under vigorous oscillation.^[^ ^302^ ^]^ This mixing initiated transformation of the 0D Cs_4_PbBr_6_ NCs to 3D perovskite CsPbBr_3_ NCs through CsBr stripping upon contact with water.^[^ ^302^ ^]^ The same surface stripping allowed silica island formation to occur on exposed surfaces asymmetrically, resulting in the final Janus geometry (Figure 8A).^[^ ^302^ ^]^ Other silicon‐containing additives have also been exploited including polyhedral oligomeric silsesquioxane (POSS), which can act as a protective matrix, enabling water resistance and prevention of anion exchange reactions (Figure 8B).^[^ ^305^ ^]^ In addition, mesoporous silica particles can be directly used to encapsulate LHP NCs to form a composite material.^[^ ^306^ ^]^ In these cases, the LHP NCs with full encapsulation in silica materials can prevent any undesired ion exchange reactions in addition to the well‐expected particle stability improvement.^[^ ^306^ ^]^

Beyond silica, polymers and lipids have also been utilized in coating LHP NCs and promote enhanced stability.^[^ ^230^, ^307^, ^308^, ^309^, ^310^, ^311^, ^312^, ^313^ ^]^ In one example, polyvinylpyrrolidone was added during LHP NC synthesis to serve as a ligand to create NCs with a PS polymer‐compatible surface.^[^ ^230^ ^]^ Following postsynthetic processing, the NCs were embedded into micro hemispheres of a PS matrix, endowing them with better water resistance.^[^ ^230^ ^]^ Long‐chain poly(maleic anhydride‐*alt*‐1‐octadecene) has also been added to enhance NC stability, and resulted in an increase in PL QY as a result of surface defect passivation.^[^ ^307^ ^]^ For a closer look at the effects that polymer matrices have on the stabilities and emission properties of LHP NCs, common polymers including PS, TOPAS (cyclic olefin copolymer), PMMA, and styrene–ethylene–butylene–styrene block copolymer were used as matrices for single particle fluorescence measurements (Figure 8C,D).^[^ ^314^ ^]^ A blue‐shift in the PL of a single particle was used as an accelerated stability test and signified deterioration of the NC at the surface.^[^ ^314^ ^]^ PS was the best polymer tested, showing no detectable blue‐shift within the tested time scale (100 s), demonstrating its promise for NC encapsulation while maintaining NC ensemble properties (Figure 8D).^[^ ^314^ ^]^ Other block copolymers including polystyrene‐*block*‐poly(ethylene‐*ran*‐butylene)‐*block*‐polystyrene and poly(ethylene glycol)‐*block*‐poly(propylene glycol)‐*block*‐poly(ethylene glycol) have been used for LHP NC shelling, which can be further coated with polyethylene glycol (PEG) molecules for biocompatibility.^[^ ^315^ ^]^ Besides direct coating of polymer precursors, a swelling/deswelling method was introduced to incorporate the LHP NCs into polymer matrices (Figure 8E,F).^[^ ^311^ ^]^ This method can also be applied to co‐encapsulate LHP NCs with magnetite nanoparticles, enabling optically and magnetically dual‐responsive composites with applications in multi‐modal biological imaging.^[^ ^310^, ^311^, ^316^ ^]^

LHP NCs have also been impregnated within other types of matrices for enhanced stability. For example, aluminum stearate, zinc stearate, and sodium stearate were introduced into a CsPbX_3_ colloidal solution, leading to the coprecipitation of the metal stearate salts with the NCs.^[^ ^317^ ^]^ Both the aluminum stearate@CsPbX_3_ and zinc stearate@CsPbX_3_ nanocomposites exhibited great water stability afforded by the insoluble nature of the metal stearate salts.^[^ ^317^ ^]^ During the impregnation process, it is hypothesized that the metal stearate binds to the surface of the LHP NCs through the formation of Cs–stearate and Pb–stearate bonds, thus replacing the native OAm ligands.^[^ ^317^ ^]^ Inorganic NaYF_4_ and ZnO have also been used to form protective capsules around CsPbBr_3_ NCs, imparting water, polar solvent, oxygen, and temperature resistance.^[^ ^318^ ^]^ Ligand concentration and reaction time were found to play a big role in control over the growth of the inorganic capsule (size of NaYF_4_ capsule increases from 50 to 500  nm with increase in OA).^[^ ^318^ ^]^

Despite the advances in LHP NC encapsulation, it should be noted that thus far it has proven challenging to achieve uniform encapsulation of LHP NCs on a single particle level in solution, thus limiting the scope of stabilization on the optical properties post‐encapsulation.

## 5 Role of Surface Ligands in Reactions with LHP NCs and LHP NC Processing

Following synthesis there are many reactions and postsynthetic processing methods that can be used on LHP NCs to render them useful for incorporation into devices. Simple addition of excess ligands to a colloidal solution of LHP NCs can trigger a perovskite phase change along with a NC morphology alteration. One of the most common types of reactions that start with LHP NCs is ion exchange, where ligands can play vital roles in the ability to tune NC composition post synthesis. For postsynthetic processing, the NCs are first assembled or transformed into hierarchical structures through a variety of techniques including self‐assembly, postsynthetic pressure processing, and NC‐based thin film formation. In all cases, these processing techniques strive to achieve interconnection between adjacent NCs while retaining or improving NC properties, in order to achieve large arrangements of material with preferred device qualities, such as conductivity. Oftentimes, long chain ligands hinder the charge transport in films, leading to the need for ligand engineering in device fabrication.^[^ ^99^ ^]^ As a result, ligands play a vital role in this process, and are required to maintain stability without serving as insulators between adjacent NCs.

### 5.1 Ligand‐Induced Structural, Morphological, and Optical Changes in LHP NCs

**Figure 9:**
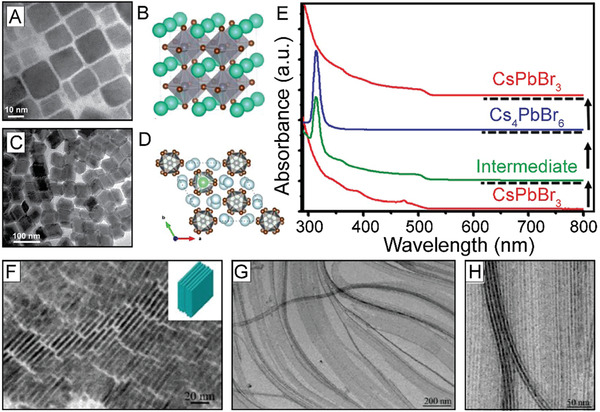
Structural and morphological changes in LHP NCs following alteration of ligand ratio post synthesis. TEM image^[^ ^321^ ^]^ A) and crystal structure schematic^[^ ^319^ ^]^ B) of as‐synthesized CsPbBr_3_ NCs. TEM image^[^ ^321^ ^]^ C) and crystal structure schematic^[^ ^319^ ^]^ D) of lead‐depleted 0D Cs_4_PbBr_6_ NCs after phase transformation of CsPbBr_3_ upon addition of excess OAm. A,C) Reproduced with permission.^[^ ^321^ ^]^ Copyright 2017, American Chemical Society. B,D) Reproduced with permission.^[^ ^319^ ^]^ Copyright 2017, American Chemical Society. E) Absorption spectra evolution during transition from CsPbBr_3_ to Cs_4_PbBr_6_ and back through changing the ratio between OA and OAm. Adapted with permission.^[^ ^321^ ^]^ Copyright 2017, American Chemical Society. F) TEM image of OA treated CsPbBr_3_ nanocubes which transformed into nanoplatelets. G,H) TEM images of CsPbBr_3_ nanocubes that were treated with small amounts of OAm to form nanowires. F–H) Reproduced with permission.^[^ ^256^ ^]^ Copyright 2019, Springer.

The most commonly used ligands in LHP NC synthesis are OA and OAm, which not only attach to the NC surface in a very labile manner, but can also go through reactions with each other, as Brönsted acids and bases.^[^ ^84^ ^]^ This spontaneous reaction combined with their dynamic nature, allows for a strong effect on LHP NC properties including absorption, PL, PL QY, crystal structure, and particle morphology, during synthesis and even postsynthesis, through alteration of ligand ratios present in the colloidal solution. The most heavily reported example is the structural transformation that CsPbX_3_ NCs can undergo to form lead‐depleted, 0D Cs_4_PbX_6_ perovskites through the addition of excess alkylamines.^[^ ^256^, ^319^, ^320^, ^321^, ^322^ ^]^ This process is initiated as a result of the ability of OAm to form a complex with PbX_2_, removing it from the cubic perovskite crystal structure. The phase transition involves complete NC dissolution of CsPbX_3_ nanocubes followed by formation of rhombohedral 0D Cs_4_PbX_6_ perovskite NCs (**Figure** **9**A–D).^[^ ^319^, ^320^, ^321^ ^]^ Formation of 0D perovskite NCs is characterized by a change in absorption features, resulting in a sharp, characteristic absorption peak (e.g., centered at 317  nm for Cs_4_PbBr_6_) and loss of PL (Figure 9E).^[^ ^320^, ^321^ ^]^ This transformation is also reversible, and transition back to cubic CsPbX_3_ NCs has been demonstrated through addition of excess OA, a mixture of PbX_2_ co‐dissolved in toluene with OA and OAm, or addition of poly(maleic anhydride‐1‐*alt*‐octadecene).^[^ ^321^, ^322^, ^323^ ^]^ Other ligand additives have also been used to improve this transformation process, including alkyl thiol ligands, which are able to bind to PbBr_2_ more strongly and precipitate out of solution, resulting in 0D perovskite NCs with improved uniformity and stability.^[^ ^319^ ^]^ Additionally, ligand additives have been used to transform cubic CsPbX_3_ NCs into other, lower‐dimensional, perovskite‐related crystal structures.^[^ ^256^, ^324^, ^325^ ^]^ For example, different amounts of alkyl thiol ligand additives can be added with either OA or OAm to trigger the formation of tetragonal CsPb_2_Br_5_ nanowires and nanosheets.^[^ ^324^ ^]^ Similarly, CsPb_2_Br_5_ nanosheets have been synthesized through a postsynthetic transformation induced by addition of dodecylammonium bromide, which initiated NC dissolution through formation of Pb_2_Br_5_^–^ complexes as an intermediate.^[^ ^325^ ^]^ In addition to the crystal structural transformation, adding smaller amounts of OAm or OA to a colloidal solution of CsPbBr_3_ nanocubes can change the morphology of the starting NCs into nanoplatelets for added OA and nanowires for added OAm without disrupting the cubic perovskite crystal structure (Figure 9F–H).^[^ ^256^ ^]^ Alternatively, ultrathin CsPbX_3_ nanowires have been formed through a transformation reaction starting from Cs_4_PbX_6_ NCs through adding a combination of short alkyl chain ligands (hexanoic acid and octylamine), OA and PbX_2_.^[^ ^326^ ^]^ In this case, PbX_2_‐ligand intermediates served as an anisotropic template for the formation of the nanowires.^[^ ^326^ ^]^

The discovery of inter‐dimensional conversion and morphological transformation triggered by change in ligand environment, on one hand, sheds additional light on the fragility of the perovskite lattice and demonstrates that caution measures must be taken to avoid NC decomposition. On the other hand, dependency on ligand composition paved the way toward a new method to achieve novel morphologies of 3D CsPbX_3_ NCs (Figure 9F–H).^[^ ^256^ ^]^ Furthermore, study of this transformation in lead‐based perovskite NCs has sparked interest in the transformation for lead‐free perovskite analogs, further expanding the library of perovskite materials.^[^ ^327^, ^328^, ^329^, ^330^ ^]^

### 5.2 Ligand Role in Postsynthetic Compositional Tuning and Doping Reactions

One of the main ways to change optical properties of LHP NCs is through changing the NC composition, which in turn changes the bandgap of the material.^[^ ^43^ ^]^ Additionally, due to the ionic structure and dynamic ligand behavior, LHP NC composition can be tuned postsynthesis through ion exchange reactions. Although halide ions (i.e., X site) are the most easily exchanged, due to the high concentrations of halide vacancies and low activation energies affording high anionic mobilities,^[^ ^39^, ^57^ ^]^ reports of ion exchange in the “A” and “B” sites have also been reported.^[^ ^56^, ^57^, ^58^, ^59^, ^60^, ^61^, ^63^, ^67^ ^––^ ^70^, ^331^, ^332^, ^333^, ^334^, ^335^ ^]^ Ligands which are present in the colloidal solution have been shown to play multiple important roles during these exchange reactions through interactions with the ion‐containing precursors to achieve activation and interactions at the NC surface.

In many examples of halide exchange, oleylammonium halide salts were used as precursors, since the NCs were typically coated with OAm species, and interruption of the native ligand shell could be largely avoided.^[^ ^57^, ^336^ ^]^ Besides oleylammonium halides, several more complex ligands have been designed to trigger halide exchange with chlorinated solvents, providing a controlled release of the halide source. In one example, triphenyl (9‐phenyl‐9*H*‐carbazol‐3‐yl)‐phosphonium bromide (TPP‐carz), was designed in order to trigger halide exchange starting from CsPbBr_3_ NCs and using the chlorobenzene solvent as a halide source.^[^ ^337^ ^]^ Specifically, TPP‐carz catalyzed carbon—chloride bond cleavage in chlorobenzene for more precise control over chloride concentrations, while simultaneously serving as a ligand.^[^ ^337^ ^]^ Poly(lactic acid) has also been used as a ligand to facilitate Br^–^ to Cl^–^ anion exchange in CsPbBr_3_ NCs using a chloride‐containing solvent as a halide source.^[^ ^338^ ^]^ Specifically, the carboxyl groups of poly(lactic acid) first bind to the Pb^2+^ in the perovskite structure, freeing H^+^ which can subsequently react with the chloroform solvent to break C—Cl bonds.^[^ ^338^ ^]^ Furthermore, the free hydroxyl group of the poly(lactic acid) can form hydrogen bonding interactions with the amine ligands and reduce their local concentrations, allowing for the Cl^–^ ions to absorb onto the NC surface more readily.^[^ ^338^ ^]^ Tetrabutylammonium *p*‐toluene sulfonate and sodium dodecylbenzene sulfonate were added to a colloidal solution of CsPbBr*_x_*Cl_3−_ *_x_* NCs to achieve either Cl‐ or Br‐rich NCs, without the need for a halogenated solvent.^[^ ^339^ ^]^ The additives instead worked by triggering an anion exchange with excess free halides present in the colloidal solution through interactions with the NC surface.^[^ ^339^ ^]^

**Figure 10:**
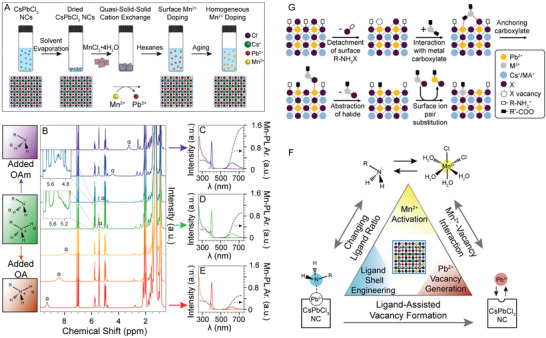
Ligand role in ion exchange reactions. A) Reaction schematic for a quasi‐solid–solid cation exchange to achieve Mn^2+^‐doped CsPbCl_3_ NCs. B) ^1^H NMR of a colloidal solution of CsPbCl_3_ NCs with added OA or added OAm. Shift in *α* peak position followed a trend with added OA and added OAm. After adding ligands, the colloidal solution was dried for cation exchange. C–E) Resulting optical spectra following the solid‐state Mn^2+^ cation exchange with C) added OAm, D) no added ligands, and E) added OA. Mn–PL Ar. is the Mn–PL peak area normalized to the bandgap PL. F) Schematic image of the mechanism of the quasi‐solid–solid cation exchange which is controlled by altering ligand composition. A–F) Adapted with permission.^[^ ^86^ ^]^ Copyright 2020, American Chemical Society. G) Schematic representation of “B” site cation exchange afforded through the use of metal carboxylate precursors, which could lead to the formation of Pb^2+^ vacancies through an anchoring mechanism. Adapted with permission.^[^ ^346^ ^]^ Copyright 2019, American Chemical Society.

In terms of “B” site cation exchange, the reaction becomes even more difficult as a result of the large activation energies required for exchange due to the high coordination number (i.e., six) of the Pb^2+^ ion within the PbX_6_ octahedra. As such, most “B” site cation exchange reaction designs have utilized ligands to promote postsynthetic partial‐ (i.e., doping) rather than full‐cation exchange. This has been especially demonstrated in Mn^2+^ doping owing to its interesting optical and magnetic properties.^[^ ^331^, ^332^, ^333^, ^334^, ^335^, ^340^, ^341^, ^342^, ^343^ ^]^ In particular, an additional Mn‐dopant emission (transition from ^6^ *A*_1g_ to ^4^ *T*_1g_ electronic states) can be introduced through transferring energy from the host LHP NCs to Mn‐dopants.^[^ ^74^, ^77^, ^344^, ^345^ ^]^ We recently demonstrated one example of a Pb^2+^‐to‐Mn^2+^ partial cation exchange for CsPbCl_3_ LHP NCs.^[^ ^86^ ^]^ The reaction was carried out on dried CsPbCl_3_ NCs, giving direct contact between ligands and the solid Mn^2+^ precursor (i.e., MnCl_2_·4H_2_O, **Figure** **10**A).^[^ ^86^ ^]^ Mn^2+^ dopant amounts could be controlled through the alteration of ligand composition, which could be monitored using ^1^H NMR (Figure 10B–E) in the initial colloidal solution before NC drying.^[^ ^86^ ^]^ Increased OAm amount allows for a higher Mn^2+^ doping concentration as a result of the interplay among ligands, the Mn^2+^ precursor identity, and the NC surface all together in a confined space (Figure 10F).^[^ ^86^ ^]^ In another example, manganese oleate complexes were reported in cation exchange reactions to achieve Mn^2+^ doping, and were proven to aid in enhancing the ability of Mn^2+^ to replace Pb^2+^ in a halide‐driven cation exchange environment.^[^ ^334^ ^]^ For other types of “B” site doping, dopant‐metal‐carboxylate precursors (i.e., bismuth 2‐ethylhexanoate, tin(II) 2‐ethylhexanoate, and zinc 2‐ethylhexanoate) were specially designed in order to yield a more successful exchange.^[^ ^346^ ^]^ The metal carboxylate salt is able to interact with the organic ammonium and halide ligands on LHP NCs, resulting in the breaking of the PbX_6_ octahedra and thus allowing for a more facile exchange between the dopants and Pb^2+^ ions (Figure 10G).^[^ ^346^ ^]^

### 5.3 Ligands in Postsynthetic LHP NC Processing and Assembly

Capping ligands play imperative roles in self‐assembly, thermal‐ and pressure‐processing, and LHP NC incorporation into films. These types of postsynthetic processing strategies take the development of LHP NCs a step further and look beyond NC behavior in colloidal solutions to focus more on ensemble behavior of NC assemblies. Furthermore, these types of studies are often closely related to device fabrication with vast impact on final device performances.

#### 5.3.1 Self‐Assembly and Pressure Processing of LHP NCs

Self‐assembly is heavily studied for conventional QDs (e.g., Cd‐chalcogenide and Pb‐chalcogenide QDs), where the QD particles are treated as “artificial atoms” to build controlled hierarchical structures. Our group has focused heavily on the self‐assembly of conventional QDs, where shape control afforded ligand patchiness leading to enhanced control over the final assembled structures, even leading to quasi‐crystalline assemblies.^[^ ^13^, ^14^, ^15^ ^]^ Furthermore, we have utilized synthetic control to yield novel heterostructural and heterocompositional QDs that helped to obtain self‐assembled superstructures with long‐range atomic alignments.^[^ ^33^, ^34^, ^347^ ^]^ However, self‐assembly of LHP NCs has mainly been limited to the assembly of nanocubes and shown to be affected by the ligand identity, more specifically the interdigitation of alkyl chains.^[^ ^138^ ^]^ In one example, LHP nanoplatelets were reported to go through a typical self‐assembly process, beginning with face‐to‐face alignment followed by formation of cuboid structures, achieved through using a shorter‐chain alkylamine and adjustment of the ligand ratio.^[^ ^348^ ^]^

**Figure 11:**
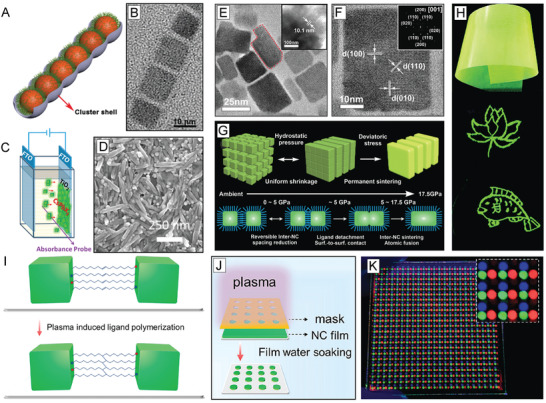
Ligand role in postsynthetic processing of LHP NCs. A) Schematic representation of nanoparticles self‐assembling to form a 1D peapod structure utilizing PbSO_4_‐oleate‐capped clusters. B) TEM image of CsPbBr_3_ NCs self‐assembled into the 1D peapod structure. A,B) Reproduced with permission.^[^ ^349^ ^]^ Copyright 2016, American Chemical Society. C) Schematic representation of the electrophoretic deposition setup used to deposit PbSO_4_‐oleate‐capped CsPbBr_3_ NC peapods onto a TiO_2_ film. D) Low magnification SEM image of a hierarchical array of electrophoretically deposited PbSO_4_‐oleate‐capped CsPbI_3_ NC peapods. C,D) Reproduced with permission.^[^ ^352^ ^]^ Copyright 2018, American Chemical Society. E) TEM image of nanoplatelets formed from putting CsPbBr_3_ NCs under pressure (inset shows TEM image of sample before nanoplatelets were dispersed in nonpolar solvent directly following pressure treatment). F) High resolution TEM image of a nanoplatelet formed during the pressure processing treatment. G) Schematic image of the pressure processing transformation process. NCs are first subjected to hydrostatic pressure, followed by formation of nanoplatelets through deviatoric stress‐induced permanent sintering during which ligands are detached and move toward the surface exposed areas of the NCs. E–G) Reproduced with permission.^[^ ^358^ ^]^ Copyright 2017, Wiley‐VCH. H) Perovskite containing films (top) and drawings (bottom) made from combining LHP NCs with silicone resins. Reproduced with permission.^[^ ^366^ ^]^ Copyright 2017, Royal Chemistry Society. I) Schematic image of ligand polymerization induced by mild plasma treatment. J) Schematic image of LHP NC film patterning through plasma treatment. K) photograph of red, green and blue NC‐dot array patterned on a 5 × 5 cm^2^ glass substrate. The diameter of each dot is 1  mm. I–​K) Reproduced with permission.^[^ ^364^ ^]^ Copyright 2019, American Chemical Society.

In other examples of self‐assembly, additives have been utilized to initiate and control the self‐assembly process. In one example, a molecular cluster‐based approach has been used to induce anisotropic assemblies of a wide variety of nanomaterials, including CsPbBr_3_ NCs.^[^ ^349^, ^350^ ^]^ Specifically, hydrophobic colloidal NCs were found to self‐assemble to form 1D superlattice chains in the presence of molecular clusters such as PbSO_4_ ligated with OAm, that could extend beyond 1  µm in length (**Figure** **11**A,B).^[^ ^349^ ^]^ The self‐assembly occurs through the formation of a polymer‐like cluster shell, which happens as a result of the strong interactions between the OA and OAm capping ligands on the NCs and the clusters (Figure 11A).^[^ ^349^ ^]^ Furthermore, simple sonication or aggregation procedures performed during the self‐assembly process were demonstrated to achieve varying assembly products (i.e., PbSO_4_‐coated NCs vs NC‐coated PbSO_4_).^[^ ^351^ ^]^ These PbSO_4_‐oleate‐capped CsPbX_3_ peapod structures have been further used to form hierarchical assemblies, where electrostatic interactions between these two materials could be utilized as a driving force to direct the assembly.^[^ ^352^ ^]^ In this example, electrophoretic deposition was utilized to form a peapod assembly on an electrode surface (TiO_2_), while preserving the linear structure of the building blocks (Figure 11C,D).^[^ ^352^ ^]^ This deposition process could be performed multiple times to form a layered film structure composed of CsPbX_3_ NCs with varying halide compositions, holding great promise for the development of LEDs and tandem‐design solar cells.^[^ ^352^ ^]^ In addition to the PbSO_4_ molecular cluster assembly strategy, unique ligands have been used to drive self‐assembly as well, like in the case of polymeric capped spherical LHP NCs, which could be assembled into pearl necklaces, bundled pearl necklaces and nanorice.^[^ ^352^, ^353^ ^]^

There are also many examples in self‐assembly where surface ligand interaction during assembly is followed by ligand desorption and relocation in order to form fused self‐assembled hierarchical structures.^[^ ^354^, ^355^, ^356^ ^]^ In this sense, nanocubes have been shown to be stitched together to form larger 3D perovskite structures or assemble in one dimension to form nanowires through an oriented attachment process.^[^ ^355^, ^356^ ^]^ Similarly, nanoplatelets can be transformed into bulk perovskite materials through a photon‐driven transformation that relies on the combined efforts of surface ligand desorption and surface ion migration.^[^ ^354^ ^]^ NC fusion has also been demonstrated in CsPbBr_3_ NCs through the use of additives, like diethylzinc.^[^ ^357^ ^]^ Although the fused LHP materials became larger and more polydispersed, the PL emission blue‐shifted and became narrower.^[^ ^357^ ^]^ This was a result of the formation of Ruddlesden‐Popper planar faults that led to efficient quantum confinement by creating deep potential wells.^[^ ^357^ ^]^ The fused NCs also exhibited higher PL QYs (77% compared to 65% for as synthesized CsPbBr_3_ NCs) due to their smaller density of surface traps as a result of their larger morphologies.^[^ ^357^ ^]^

Sintering in LHP NCs has also been demonstrated utilizing pressure processing techniques where LHP nanocubes were exposed to high pressure (between ≈2 and 18  GPa).^[^ ^358^, ^359^, ^360^, ^361^ ^]^ In one example we demonstrated that during the pressure treatment process, inorganic CsPbBr_3_ NCs came close together and ligand detachment occurred as a result of either hydrostatic or deviatoric stress induced by external pressure, ultimately allowing direct contact between surfaces of neighboring NCs.^[^ ^358^ ^]^ Additional pressure leads to NC sintering, resulting in larger nanoplatelets which exhibited PL enhancement and longer PL LTs as a result of less surface and crystalline defects, rendering the nanoplatelets promising for device incorporation (Figure 11E–G).^[^ ^358^ ^]^ Unlike their inorganic counterparts, HOIP MAPbBr_3_ NCs were found to go through a recrystallization process following exposure to elevated pressures.^[^ ^360^ ^]^ Specifically, the NCs were first comminuted into nanoslices at which point detachment of surface ligands, amorphization, and sintering led to recrystallization to form large nanoplates.^[^ ^360^ ^]^

#### 5.3.2 Formation of LHP NC Thin Films

Arguably most research effort for LHP NCs in postsynthetic processing has been devoted toward the formation of films that can be integrated into devices and/or impart additional properties. There are many considerations in terms of ligand type to be used during this process, as the ligands must promote NC stability during film fabrication but must also not be too insulating as to lead to poor device performance. In the simplest film formation examples, NCs are used directly as synthesized, without altering the ligands. For example, in one of the most straight‐forward methods for film formation, assembled LHP NC thin films were directly deposited on a glass substrate during the purification process by adding the substrate during centrifugation.^[^ ^362^ ^]^ Following the formation of OA/OAm‐capped LHP NC thin films, annealing processes commonly utilized in device fabrication methods have also been explored.^[^ ^363^ ^]^ Upon annealing an LHP NC film at 100  °C, some of the ligands were removed, while annealing at 150  °C led to a coalescence of the NCs. Overall, this change in the film structure led to a stabilized current.^[^ ^363^ ^]^ Enhanced stability of the films fabricated with as‐synthesized LHP NCs has also been demonstrated through polymerization of the double bonds on the already‐present OA and OAm ligands (Figure 11I).^[^ ^364^ ^]^ This could be achieved by mild plasma irradiation, a process that could be precisely controlled to form patterns (Figure 11J,K).^[^ ^364^ ^]^ Effects of oxygen plasma treatment on the native organic ligand shells for both CsPbX_3_ and MAPbX_3_ NCs have also been explored.^[^ ^365^ ^]^ Specifically, the O_2_ plasma mainly affects the top few nanometers of the NC film and is able to partially remove residual organics (ligands) that could be detrimental to device performances.^[^ ^365^ ^]^ Films formed from MAPbBr_3_ NCs were destroyed following O_2_ plasma treatment, evidenced from loss of PL and quenching of its electrical transport properties.^[^ ^365^ ^]^ Films formed from CsPbBr_3_ NCs, on the other hand, demonstrated enhanced stability and only partial quenching of the PL (≈50% of initial PL QY maintained following 15 min of O_2_ plasma treatment at 50 W).^[^ ^365^ ^]^

LHP NCs with native ligand shells have also been combined with other additives to form thin films.^[^ ^95^, ^313^ ^]^ Films of MAPbX_3_ NCs have been made by combining the LHP NCs with commercially available silicone resins, which allowed for increased stability against water and UV light, and could be molded into thin films, or used to create other perovskite‐containing structures (Figure 11H).^[^ ^366^ ^]^ Addition of a polyvinylpyrrolidone matrix to silicon resins through electrospinning afforded films that could not undergo anion exchange reactions, paving the way toward white emission LEDs.^[^ ^367^ ^]^ Electrospinning has also been used to encapsulate LHP NCs into hydrophobic polymers like PMMA or PS, forming fibers that could be incorporated into devices.^[^ ^368^, ^369^ ^]^ Polymerizable vinylbenzyl ammonium lead bromide has been used as a structure‐directing ligand to form MAPbBr_3_ NC films.^[^ ^370^ ^]^ Additionally, bulk polymers were used to encapsulate LHP NCs, directly forming films, and offering additional protection from solvent access, rendering them more stable toward light and water degradation.^[^ ^312^, ^313^ ^]^

In other examples, ligands or solvents utilized during NC synthesis were altered in order to impart NC films with desirable properties, including efficient charge transfer or luminescence.^[^ ^187^, ^231^, ^371^ ^]^ For instance, lauryl methacrylate monomer was used as a solvent during NC synthesis instead of ODE.^[^ ^231^ ^]^ This solvent provided the ability to complete radical polymerization and crosslinking between adjacent NCs following synthesis and addition of an initiator.^[^ ^231^ ^]^ Moreover, small organic molecules tetraphenylphosphonium bromide and ethane‐1,2‐diylbis(triphenylphosphonium) bromide were used to make a MAPbBr_3_ film where the NCs were formed in situ due to different crystallization rates between the NCs and the phosphonium matrix.^[^ ^372^ ^]^

The use of short chain ligands in nanoinks for film formation has been heavily studied and resulted in enhanced film conductivities due to the less insulating nature of the shorter ligands and the quick drying process of the inks.^[^ ^99^, ^187^, ^371^ ^]^ A room temperature synthesis method to create better inorganic LHP NC inks was developed using short‐chain, low‐boiling‐point solvents and ligands including isopropanol, hexane, propionic acid, and butylamine.^[^ ^371^ ^]^ The use of short‐chain ligands circumvented the need for any postsynthetic treatments, allowing the inks to be directly applied to make high quality films for solar cell applications.^[^ ^371^ ^]^ The use of these shorter‐chain ligands has also been studied in terms of annealing temperature, as annealing is a common practice in device fabrication. The annealing process is linked to the desorption of surface ligands which can cause instability in the LHP NC structure. When LHP NCs were capped with shorter chain ligands, NC transformation was observed at elevated temperatures, resulting in partial conversion to the tetragonal CsPb_2_Br_5_ phase.^[^ ^373^ ^]^ Although shorter chain ligands are ideal for enhanced film conductivities, a balance must be reached between NC stability and improvement of charge transport properties.

Following NC film formation, there are many reported methods aiming to further improve film properties and stability. Through studies on LHP NC thin films, it was found that the PL QY of the film could be improved post‐fabrication by ligand engineering through addition of Lewis bases in order to reduce defect densities.^[^ ^374^ ^]^ For example, fluoroalkyl trichlorosilanes have been added to a pre‐formed perovskite NC films through a vapor phase, rendering the film hydrophobic.^[^ ^375^ ^]^ This created a barrier against water and humidity, allowing for enhanced stability compared to uncoated films when exposed to water (PL intensity reduced only 30% following exposure to water for 120 min compared to 80% for the uncoated film).^[^ ^375^ ^]^

## 6 Ligand Consideration for LHP NC‐Based Applications

LHP NCs have been widely utilized in a spectrum of devices and applications including LEDs,^[^ ^376^, ^377^ ^]^ solar cells,^[^ ^378^, ^379^ ^]^ solar concentrators,^[^ ^77^ ^]^ lasing,^[^ ^51^, ^380^, ^381^ ^]^ scintillators,^[^ ^382^ ^]^ photoarrays,^[^ ^383^ ^]^ photodetectors,^[^ ^384^, ^385^, ^386^, ^387^ ^]^ catalysis,^[^ ^220^, ^246^, ^388^, ^389^, ^390^ ^]^ etc.^[^ ^213^, ^391^, ^392^ ^]^ In nearly all cases, ligands have been found to play an important role in determining device performances.^[^ ^393^ ^]^ Thus, there remains a lot of room to study how ligands affect the photophysical characteristics of NC solids for device applications.^[^ ^180^ ^]^ Although there are many great reports encompassing the incorporation LHP NCs into devices, this review will focus on studies that have directly investigated ligand roles in device performances and stability with a more in‐depth focus on LED and display applications. For a more comprehensive look at LHP NC sensitized devices and applications, we refer readers to other excellent review articles.^[^ ^38^, ^48^, ^394^ ^]^

### 6.1 Integration of LHP NCs in LEDs

The superior optoelectronic properties, including high PL QYs, render LHP NCs ideal for LED applications. Specifically, LHP NCs are typically incorporated into an emitter layer of an LED through the formation of a thin film, where both charge transport and film quality are vital to the overall device performance. Ligands play an important role in charge transport, as long carbon chain length ligands are insulating in nature and can prevent efficient transport. As such, utilizing shorter chain ligands or ligand density control are common strategies to alleviate these issues. However, stability of the LHP NCs in the film and passivation of surface defects to maintain high film QYs are also essential for device performances. Thus, a balance must be reached between charge transport and film optical performance in order to achieve optimal external quantum efficiencies (EQEs). This section will cover the ligands of varying functionalities that have been used in LED device fabrication, purification procedures that have been developed to control ligand densities, and postsynthetic film treatments that have been utilized to improve LED device performances. We will also discuss the ligand role in the use of LHP NCs for the fabrication of downconversion white LEDs (WLEDs).

#### 6.1.1 Altering the Ligand Identity in LHP NC‐Based LED Devices

In the early stages of LHP NC use in the engineering of LEDs, native alkylamine and alkyl carboxylic acids were typically kept on NC surfaces during device fabrication. The first example of an inorganic LHP NC‐based LED was fabricated in 2015 (indium tin oxide (ITO)/poly(ethylenedioxythiophene):polystyrene sulfonate (PEDOT:PSS)/poly(9‐vinylcarbazole) (PVK), CsPbX_3_ NCs/1,3,5‐tris(*N*‐phenylbenzimidazole‐2‐yl)benzene (TPBi)/LiF/Al), and showed a maximum luminance of 946  cd m^−2^ at an applied voltage of 8.8  V with an EQE of 0.12%.^[^ ^376^ ^]^ Beyond this initial study, ligand identity has been altered to further improve device performances.

##### Effect of Alkylamine and Alkyl Carboxylic Acid Chain Length on LED Performance

**Figure 12:**
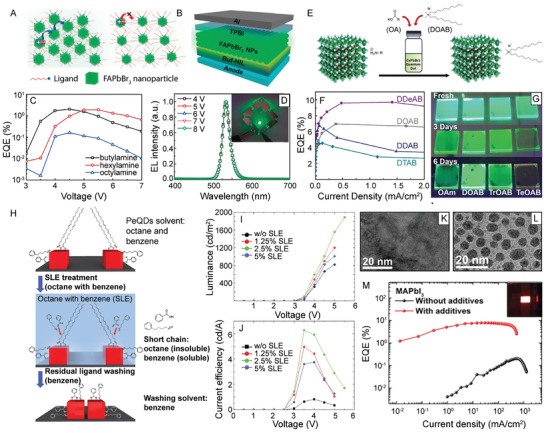
LHP NCs in LEDs. A) Schematic image of the efficiency of charge injection and transport in LHP NC films formed with FAPbBr_3_ NCs passivated with short carbon chain (left) and long carbon chain (right) ligands. B) Schematic image of the LED device fabricated using FAPbBr_3_ NCs. C) EQE versus voltage of LEDs fabricated using FAPbBr_3_ NCs passivated with butylamine (black), hexylamine (red), and octylamine (blue). D) EL spectra of LEDs fabricated with butylamine‐passivated FAPbBr_3_ NCs at different applied biases. Inset shows an image of the LED device. A–D) Adapted with permission.^[^ ^395^ ^]^ Copyright 2017, Elsevier. E) Schematic image of the ligand exchange process using quaternary alkylammonium halides. F) EQE versus current density for LEDs fabricated using CsPbBr_3_ NCs passivated with quaternary alkylammonium bromide ligands of varying carbon chain length. G) Photographs of thin films formed from CsPbBr_3_ NCs passivated with quaternary alkylammonium bromide ligands of varying length under UV illumination over time. E–G) Reproduced with permission.^[^ ^400^ ^]^ Copyright 2019, American Chemical Society. H) Schematic image of a solid‐state ligand exchange (SLE) process to achieve benzoic acid and 4‐phenylbutylamine capped LHP NCs in the NC film. I) Luminance versus voltage for LHP NC‐based LED devices that underwent different SLE conditions. J) Current efficiency versus voltage for LHP NC‐based LED devices that underwent different SLE conditions. H–​J) Adapted with permission.^[^ ^412^ ^]^ Copyright 2018, American Chemical Society. K) Cross‐sectional TEM image of a MAPbI_3_ thin film without 4‐fluorophenylmethylammonium iodide additives. L) Cross‐sectional TEM image of a MAPbI_3_ thin film with 4‐fluorophenylmethylammonium iodide additives showing in situ growth of MAPbI_3_ NCs during the film formation process. M) EQE versus current density of MAPbI_3_ LEDs with (red) and without (black) 4‐fluorophenylmethylammonium iodide additives. K–M) Adapted with permission.^[^ ^419^ ^]^ Copyright 2017, American Chemical Society.

In LHP NC‐based LEDs, charge injection and transport capability were improved through adjusting the length of the alkylamine and alkyl carboxylic acid ligands, either during the LHP NC synthesis or through ligand exchange strategies.^[^ ^252^, ^290^, ^395^ ^]^ For example, alkylamines with varying carbon chain lengths (i.e., *n*‐butylamine, *n*‐hexylamine, *n*‐octylamine) were used in the direct synthesis of FAPbBr_3_ HOIP NCs, which were then further utilized in the fabrication LED devices (**Figure** **12**A–D).^[^ ^395^ ^]^ First, to characterize the charge injection and transport capabilities of the NC films formed from the different passivated FAPbBr_3_ NCs, a hole‐only device was fabricated where the current density increased as the length of the amine ligand decreased, indicating that the use of a shorter‐chain ligand can improve the electroluminescence (EL) efficiency by improving charge injection and transport capabilities within the LHP NC film (Figure 12A).^[^ ^395^ ^]^ This was further demonstrated through the fabrication of simplified LEDs (ITO/Buf‐HIL/FAPbBr_3_ NCs/TPBi/LiF/Al) (Figure 12B), where the current density increased and the turn‐on voltage decreased upon shortening the carbon chain length of the amine ligand.^[^ ^395^ ^]^ Additionally, the EQE increased from *n*‐octylamine to *n*‐butylamine, reaching a maximum value of ≈2.05%, demonstrating ligand engineering as an effective means to enhance device performances (Figure 12C).^[^ ^395^ ^]^ The fabricated LED devices also showed stable EL spectra (*λ* ≈ 530  nm) under different applied biases with the capability to fulfill a pure green color in the national television system committee (NTSC) chromaticity diagram (Figure 12D).^[^ ^395^ ^]^ The library of *n*‐alkylamine ligands probed for LED device applications was recently expanded, varying from *n*‐ethylamine to *n*‐heptylamine for MAPbBr_3_ NC‐based LED devices.^[^ ^252^ ^]^ Briefly, fabricated LEDs (ITO/PEDOT:PSS/poly(4‐butylphenyl‐di‐phenyl‐amine) (poly‐TPD)/MAPbBr_3_ NCs/TPBi/lithium 8‐quinolate (Liq)/Al) with *n*‐butylamine through *n*‐heptylamine ligands demonstrated favorable luminance‐voltage characteristics and low‐turn on voltages (3.4–3.5  V).^[^ ^252^ ^]^ However, unlike the previous example, devices fabricated with the NCs possessing the longest carbon chain‐length ligand, *n*‐heptylamine, showed the greatest EQE of 1.5%.^[^ ^252^ ^]^ This increase in EQE following an increase in ligand chain length was attributed to the superior film qualities and the high PL QY of the LHP NC film.^[^ ^252^ ^]^ The importance of film quality was further demonstrated when butylamine was used along with short chain propionic acid in a low‐temperature synthesis of CsPbBr_3_ NCs for directly fabricating LED devices (ITO/PEDOT:PSS/CsPbX_3_ NCs/TPBi/Ca/Ag), which failed to work due to poor film quality .^[^ ^290^ ^]^ In order to improve the LED device performances, ligand exchange reactions were performed and LEDs fabricated from OA and OAm passivated CsPbBr_3_ NCs exhibited the best device performances (maximum luminance of 5033  cd m^−2^, current efficiency of 18.6  cd A^−1^, and EQE of 5.4%) despite possessing ligands with the longest carbon‐chain length (C18) tested.^[^ ^290^ ^]^ This improvement in device performance was attributed to the NC film quality and superior defect passivation, which was supported by lengthened PL LTs that led to enhanced device luminance.^[^ ^290^ ^]^ Together, these findings all demonstrate that a balance between NC quality (e.g., stability, defect passivation, and film fabrication) and the insulating nature of the ligands (e.g., charge injection and charge transport) must be reached for improved LED device performances.^[^ ^180^, ^252^, ^290^, ^395^ ^]^

##### Effect of Alkylamine and Alkyl Carboxylic Acid Bulkiness and Aromaticity on LED Performance

The effect of other, more sterically hindered or aromatic amine ligands on LHP NC‐based devices has also been explored. For example, an aromatic amine ligand, 3‐phenyl‐2‐propen‐1‐amine, has been used as a ligand for CsPbBr_3_ NCs incorporated into LED devices (ITO/PEDOT:PSS/poly‐TPD/CsPbBr_3_ NCs/TPBi/LiF/Al).^[^ ^396^ ^]^ The resulting LED showed a maximum EQE of 7.13% as a result of increased carrier transport due to electron delocalization and *π* − −*π* stacking afforded from the ligands.^[^ ^396^ ^]^ Mixed halide CsPbCl*_x_*Br_3−_ *_x_* NCs treated with adamantane‐1,3‐diamine to achieve ligand exchange, were incorporated into blue emitting (*λ* = 456  nm) LEDs (ITO/PEDOT:PSS/poly(9,9‐dioctyl‐fluorene‐*co*‐*N*‐(4‐butylphenyl)‐diphenylamine) (TFB)/CsPbCl_x_Br_3−_ *_x_* NCs/ TPBi/Liq/Al) with a demonstrated EQE of 0.49% .^[^ ^397^ ^]^ For a further improvement of EQE, oleylammonium chloride was also incorporated into the LED device as an interfacial layer to reduce the current density, resulting in a more than doubled EQE of 1.1%.^[^ ^397^ ^]^ In addition to sterically hindered amine ligands, adamantane carboxylic acid has been added to colloidal solutions of MAPbI_2_Br NCs to enhance the PL QY.^[^ ^398^ ^]^ LED devices fabricated using these NCs (ITO/PEDOT:PSS/poly‐TPD/MAPbI_2_Br NCs/TPBi/LiF/Al) possessed an EL peak centered at 635  nm with an EQE of 2.75%.^[^ ^398^ ^]^ However, at practically relevant current densities, the device suffered from halide segregation issues that are commonly observed for devices fabricated using LHP NCs with mixed Br/I halide compositions.^[^ ^398^ ^]^

##### Quaternary Alkylammonium Halide Passivated LHP NCs in LED Devices

Quaternary alkylammonium halides are sterically bulky ammonium ligands that have been heavily utilized to passivate LHP NCs when forming films for LED devices. Specifically, DDAB has been chosen as a relatively short ligand and utilized to improve charge carrier transport.^[^ ^189^, ^399^ ^]^ When DDAB‐passivated CsPbBr_3_ NCs were first incorporated into an LED device, (ITO/PEDOT:PSS/PVK/CsPbBr_3_ NCs/TPBi/LiF/Al), a maximum luminance of 330  cd m^−2^ and an EQE of 3.0% was reported.^[^ ^399^ ^]^ Additionally, the turn‐on voltage for the device was 3.0  V, which was lower than the LEDs fabricated using CsPbBr_3_ NCs with native OA/OAm ligands.^[^ ^399^ ^]^ This indicated that efficient and barrier‐free charge injection was achieved following the ligand exchange process to yield DDAB‐passivated NCs.^[^ ^399^ ^]^ DDAC/DDAB ligands have also been used to achieve more efficient blue emission in mixed halide (Br and Cl) LHP NC‐based LEDs.^[^ ^188^, ^286^, ^399^ ^]^ For example, DDAC/DDAB‐capped CsPbCl*_x_*Br_3−_ *_x_* NCs incorporated into an optimized LED device structure (i.e., ITO/PEDOT:PSS/poly[*N*,*N*′‐bis(4‐butylphenyl)‐*N*,*N*′‐bisphenylbenzidine] (pTPD)/PVK/CsPbCl*_x_*Br_3−_ *_x_* NCs/B2PYMPM/TPBi/LiF/Al) yielded high EQEs of 1.03%, 2.25%, 3.50%, 4.96%, and 9.80% for EL spectra centered at 463, 476, 490, 502, and 512  nm, respectively.^[^ ^188^ ^]^ Chain length and bulkiness of other quaternary​ alkylammonium bromides have also been explored in order to gain an understanding of the effect of ligand composition on device performances.^[^ ^186^, ^187^, ^400^ ^]^ In one study, an expanded ligand library of quaternary alkylammonium halides was tested using a ligand exchange process on OA/OAm capped CsPbBr_3_ NCs (i.e., dioctyldimethylammonium bromide (DOAB), methyltrioctylammonium bromide, tetraoctylammonium bromide, didecyldimethylammonium bromide (DDeAB), DDAB, and ditetradecyldimethylammonium bromide) (Figure 12E).^[^ ^400^ ^]^ The NCs were then applied to fabricate LED devices (ITO/PEDOT:PSS/poly‐TPD/CsPbBr_3_ NCs/LiF/Al), and better optical stability and properties were achieved in the LEDs using LHP NCs with less bulky ligands and ligands with shorter carbon chain lengths.^[^ ^400^ ^]^ Through tuning the ligand steric hindrance, a maximum luminance of 2269  cd m^−2^ and an EQE of 9.7% were achieved for LEDs fabricated with DDeAB‐capped CsPbBr_3_ NCs (Figure 12F).^[^ ^400^ ^]^ Surprisingly, the ligand with the shortest chain length of its carbon chain branches (DOAB) had a slightly worse performance than DDeAB (Figure 12F).^[^ ^400^ ^]^ This was attributed to DDeAB having a better charge‐carrier balance for radiative recombination in the device set‐up compared with DOAB.^[^ ^400^ ^]^ Overall, dependence of device properties on ligand identity was accredited to both the increase in NC trap sites upon increase in ligand bulkiness and the decrease in charge transport upon increase in ligand carbon chain branch length.^[^ ^400^ ^]^ NC film stability in air with a relative humidity of 60% was also studied over time for CsPbBr_3_ NCs capped with quaternary alkylammonium halide ligands possessing an increasing number of octyl‐carbon chain branches (i.e., DOAB, methyltrioctylammonium bromide, and tetraoctylammonium bromide), and all films tested showed a weakened PL intensity as time passed (Figure 12G).^[^ ^400^ ^]^ The DOAB ligand, with the smallest steric hindrance of the tested quaternary alkylammonium ligands provided the most stable PL emission following six days.^[^ ^400^ ^]^ However, improving film stability when using LHP NCs with quaternary alkyl ammonium halides as ligands still stands as a challenge.

##### Other Ligands, Additives, and Encapsulation Strategies Used to Passivate LHP NCs in LEDs

Beyond the traditional amine‐ and ammonium‐based ligand coverage, ligands with varying functional groups have been used to passivate LHP NCs incorporated into LED devices. For example, octylphosphonic acid has been used to replace native OA and OAm ligands in CsPbX_3_ NCs,^[^ ^91^, ^401^ ^]^ with the fabricated LEDs achieving EQEs as high as 7.74% (CsPbBr_3_ NCs)^[^ ^91^ ^]^ and 12.6% (CsPbI_3_ NCs).^[^ ^401^ ^]^ Bidentate 2,2′‐iminodibenzoic acid yielded enhanced electronic coupling between the ligands and NCs, which was observed in fabricated LEDs (ITO/PEDOT:PSS/poly‐TPD/CsPbI_3_ NCs/TPBi/LiF/Al) that demonstrated twofold higher EQEs and luminance intensities than those of the devices fabricated using NCs possessing native ligand coverage.^[^ ^212^ ^]^ In addition, zwitterionic sulfobetaine passivated CsPbCl*_x_*Br_3−_ *_x_* NC‐based LED devices (ITO/PEDOT:PSS/poly‐TPD/4,4′‐bis(*N*‐carbazolyl)‐1,1′‐biphenyl (CBP)/CsPbCl*_x_*Br_3−_ *_x_* NCs/4,6‐bis(3,5‐di(pyridine‐3‐yl)phenyl)‐2‐methylpyrimidine (B3PYMPM)/LiF/Al) possessed a blue emission peak centered at 463  nm with a maximum EQE of 1.2%.^[^ ^402^ ^]^ Polymerizable conjugated linoleic acid was used as a ligand in the synthesis of CsPbI_3_ NCs and was subsequently crosslinked to form LHP NC films for LED devices (ITO/PEDOT:PSS/poly‐TPD/CsPbI_3_ NCs/TPBi/LiF/Al), resulting in a maximum EQE of 2.67%.^[^ ^403^ ^]^

In some cases, ligands are selected to improve charge injection properties into the NC phosphor layer and improve NC film quality.^[^ ^404^ ^]^ Explicitly, the morphology and electronic properties of the NC film are critical to the performance of an LED device.^[^ ^200^ ^]^ For example, 1,8‐octyldiamine bromide salt (BOABr_2_) was used to passivate CsPbBr_3_ NCs, as the two terminal amines could serve as a bridge between adjacent NCs in the film.^[^ ^200^ ^]^ The resulting film had a root mean square (RMS) roughness of 3.72  nm compared to 4.02  nm for the film with native ligands, and also demonstrated enhanced charge transport.^[^ ^200^ ^]^ These BOABr_2_‐passivated NCs were further utilized in LED devices (ITO/PEDOT:PSS/modified poly (bis(4‐phenyl)(2,4,6‐trimethylphenyl)amine)/CsPbBr_3_ NCs/TPBi/LiF/Al) and demonstrated greater current density, luminance and current efficiency compared to the control (NCs capped with OAm and propionic acid) as a result of the reduced vacancy traps and enhanced charge transport.^[^ ^200^ ^]^ The carbon chain lengths on the diamine bromide salts were also altered, and the 1,4‐butanediamine bromide (C4) ligand had the best EQE of 8.56% as a result of its superior charge transport properties.^[^ ^200^ ^]^

Apart from traditional ligands, as discussed in Section  4.1, other types of additives are incorporated in the LHP NC synthesis or the colloidal solution to improve the NC quality or to control the NC surface, which, in turn, improves the LHP‐NC‐based LED device performances.^[^ ^135^, ^285^, ^405^ ^]^ In one example, excess FABr was added in the synthesis of FAPbBr_3_ NCs to passivate surface defects and enhance the PL QY.^[^ ^405^ ^]^ This ultimately led to an improved LED device (ITO/PEDOT:PSS/PVK/FAPbBr_3_ NCs/TPBi/LiF/Al) performance with a current efficiency of 76.8  cd A^−1^ and an EQE of 17.1%.^[^ ^405^ ^]^ Alternatively, heterostructure formation can work to passivate surface defects and improve device performances. For example, an inorganic PbS shell was used to cap CsPbI_3_ NCs and the resulting heterostructures were employed to fabricate LEDs with p–i–n (construction of a semiconductor device with p‐type, intrinsic, and n‐type layers) structures (ITO/ZnO/ CsPbI_3_ NCs/4,4′,4′′‐tris(carbazole‐9‐yl)‐triphenylamine (TCTA)/MoO_3_/Au).^[^ ^277^ ^]^ The resulting LEDs exhibited a turn‐on voltage of 1.78  eV, close to the NC bandgap, indicating that a barrier‐free charge injection was accomplished.^[^ ^277^ ^]^ Furthermore, a maximum luminance of 1050  cd m^−2^ was reached at 6.7  V along with improved device stability as a result of the surface stabilization introduced by the inorganic PbS shell.^[^ ^277^ ^]^

Other encapsulation strategies have also been explored and used to improve device performances in LHP NC‐based LEDs, and have been specifically demonstrated to enhance device stabilities.^[^ ^229^, ^406^ ^]^ For example, the effects of the film deposition method and NC incorporation into a polymer (i.e., PMMA) were studied.^[^ ^406^ ^]^ Drop‐casting the NCs together with PMMA prevented NC aggregation and provided a better surface coverage over other film formation methods tested such as spin coating, or drop‐casting in the absence of PMMA.^[^ ^406^ ^]^ LED devices fabricated using these PMMA‐NC films demonstrated improved long‐term stability (luminance values decreased by only ≈50% following 50 min continuous operation at a constant current density of 40.7  mA cm^−2^) and a maximum EQE of 0.25% was achieved with suppressed EL blinking for CsPbBr_3_ NC‐based devices.^[^ ^406^ ^]^

##### NC Purification Procedures to Reduce Ligand Density

In addition to the vast study of the ligand identity effects on LED device performance, purification procedures, specifically as a means to further control ligand density and improve charge injection and transport in LED devices, have been studied for a variety of the ligand types discussed in the previous sections (Sections  3 and  4). Specifically, control over ligand density to minimize the concentration of insulating ligands within a LHP NC film is one method to improve LED device performances.^[^ ^407^, ^408^, ^409^, ^410^ ^]^ For example, it was found that a mixture of ethyl acetate and hexanes could be used to lower ligand density for OA/OAm‐capped CsPbBr_3_ NCs while maintaining stability during the NC purification process.^[^ ^408^ ^]^ A maximum EQE of 6.27% has been reported from a LED device (ITO/PEDOT:PSS/poly‐TPD/CsPbBr_3_ NCs/TPBi/LiF/Al) using the CsPbBr_3_ LHP NCs with two cycles of purification.^[^ ^408^ ^]^ This reported EQE was around a 50‐fold enhancement from the devices fabricated with CsPbBr_3_ NCs that had undergone only one round of purification with acetone.^[^ ^408^ ^]^ Purification of DDAB‐passivated NCs has been also been demonstrated, and the use of polar solvents (e.g., methanol, isopropyl alcohol, and ethanol) in washing steps were proved to be detrimental to NC stability, and resulted in complete quenching of the NC emission, while butyl acetate, ethyl acetate and butanol were more benign for washing the LHP NCs.^[^ ^409^ ^]^ Specifically, the dielectric constant of the poor solvent used in purification can directly affect the PL QY, with a trend of decreasing PL QY observed with an increasing dielectric constant.^[^ ^410^ ^]^ Careful choice in the poor washing solvent aided in removal of excess free ligands while maintaining NC integrity and resulted in LEDs with a higher EQE around 8%.^[^ ^409^, ^410^ ^]^ Beyond simple purification methods, films fabricated with DDAB‐coated LHP NCs have been studied in even further detail.^[^ ^411^ ^]^ DDAB was found to hinder coalescence of NCs during annealing and photoactivation steps, further proving that DDAB is a superior ligand for LHP NC integration in LEDs.^[^ ^411^ ^]^ Overall, both careful ligand selection and purification procedures are important for improving the performances of LHP NC‐based LEDs. Although long‐chain organic ligands can be detrimental to charge transport and charge carrier injection, a delicate balance between reducing ligand density and maintaining NC stability can result in the best device performances.

#### 6.1.2 Ligand Modification in NC Films for LED Device Improvement

Another strategy for improving LED device performance is to alter ligand coverage of the NCs following the film formation process. Specifically, there are reports of solid‐state ligand exchange strategies,^[^ ^412^ ^]^ NC film post‐processing techniques,^[^ ^413^ ^]^ and in situ NC formation during the film fabrication process.

##### LHP NC Film Treatments

Solid‐state ligand exchange is an alternative strategy to adjust the ligand shell composition of the LHP NCs in films.^[^ ^412^, ^414^, ^415^ ^]^ This type of ligand exchange is especially promising for LHP NCs which, due to the labile nature of the native OA and OAm ligands, could be affected by solubility and stability issues in a traditional solution‐phase ligand exchange strategy.^[^ ^412^ ^]^ In a solid‐state ligand exchange, the new ligand is dispersed in an antisolvent, which is then dropped onto the surface of a NC film (Figure 12H).^[^ ^412^, ^414^, ^415^ ^]^ However, due to the sensitivity of LHP NCs to nonpolar solvents, careful solvent choice is required to accomplish the ligand exchange process.^[^ ^412^ ^]^ In one example of a solid‐state ligand exchange performed on a LHP NC film, short chain ligands such as benzoic acid and 4‐phenylbutylamine in a mixture of octane and benzene were used to replace native OA and OAm ligands (Figure 12H).^[^ ^412^ ^]^ Different concentrations of the short‐chain ligands in the precursor solution were altered, and the device performances were compared.^[^ ^412^ ^]^ The luminance was found to increase as the concentration of the short‐chain ligand was increased (Figure 12I).^[^ ^412^ ^]^ Specifically, the device luminance increased from 821  cd m^−2^ for the film with native ligands to 1889  cd m^−2^ for the film that underwent the solid‐state ligand exchange with the solution containing 2.5% short chain ligands (i.e., 4‐phenylbutylamine and benzoic acid), demonstrating a 230% enhancement.^[^ ^412^ ^]^ The current efficiency was also improved upon solid‐state ligand exchange, with the best results also observed for the film that underwent the exchange with the ligand solution containing 2.5% short chain ligands (i.e., 4‐phenylbutylamine and benzoic acid, Figure 12J).^[^ ^412^ ^]^

Post‐processing LHP NC films without exchanging ligands can also improve the device performances of the resulting LEDs, such as through enhancing device stabilities.^[^ ^413^ ^]^ For example, a trimethylaluminum (TMA) crosslinking technique was developed to stabilize the NC film for further device processing.^[^ ^413^ ^]^ Specifically, native ligands allow CsPbX_3_ NC solubility in a variety of organic solvents, e.g., chlorobenzene, toluene, etc., which limits solvents that can be used in subsequent device fabrication steps to avoid dissolution of the perovskite layer. The TMA crosslinking technique allows for the NC film to be fixed in place, ensuring film stability throughout the entirety of the LED assembly.^[^ ^413^ ^]^ Devices that underwent this NC film post processing procedure possessed an EQE as high as 5.7%, which was an order of magnitude greater than their non‐crosslinked counterparts.^[^ ^413^ ^]^

##### In Situ LHP NC Formation in LED Device Fabrication Methods

In some cases of fabricating LHP NC‐based LED devices the LHP NCs are synthesized via an in situ process during film formation.^[^ ^372^, ^416^, ^417^, ^418^, ^419^, ^420^, ^421^ ^]^ In this method, the choice of ligands is critically important to promote NC formation during the film fabrication process. In the process, a film is typically produced using a spin‐coating technique where NC precursors are dispersed in a good solvent, and NCs are formed upon solvent evaporation, or through subsequent addition of a bad solvent.^[^ ^372^, ^418^, ^419^ ^]^ In one of the earliest examples, small molecule 2,2′,2′′‐(1,3,5‐benzinetriyl)‐tris(1‐phenyl‐1*H*‐benzimidazole) was added to control and reduce MAPbBr_3_ grain size (100–250  nm), and resulted in highly bright and efficient LEDs (self‐organized conducting polymer (SOCP)/MAPbBr_3_ NCs/TPBi/LiF/Al) with an EQE of 8.53%.^[^ ^416^ ^]^ Later, NC size could be further decreased through changing the identity of the organic ligands, typically focusing on ligand bulkiness.^[^ ^372^, ^417^, ^418^, ^419^, ^420^, ^421^ ^]^ For example, *n*‐butylammonium halide ligands have been incorporated into precursor solutions to occupy “A” sites in the perovskite structure, thus preventing crystallite growth beyond the nanoscale.^[^ ^417^ ^]^ As a result, the EQE of MAPbI_3_‐incorporated LEDs increased from 1.0% to 10.4% upon introduction of n‐butylammonium iodide.^[^ ^417^ ^]^ In another example, bulky 4‐fluorophenylmethylammonium iodide (FPMAI) was used in MAPbI_3_ film fabrication.^[^ ^419^ ^]^ Addition of FPMAI resulted in the formation of ≈5.4  nm NC grains (Figure 12L), which were not seen without the ligand additive (Figure 12K).^[^ ^419^ ^]^ The EQE versus current density curve of the LED device (ITO/poly‐TPD/MAPbI_3_ NCs/TPBi/LiF/Al) with FPMAI additive showed an enhanced EQE of 7.9% compared to 0.2% for the film formed without additive (Figure 12M).^[^ ^419^ ^]^

Beyond altering ligand identity, additives and matrices have been utilized to further enhance device performances for the LEDs with in situ fabricated LHP NCs.^[^ ^422^, ^423^ ^]^ As LHP NC quality plays a vital role in LED performance, strategies to enhance PL efficiencies have been demonstrated through the addition of additives during fabrication of the NC films.^[^ ^405^ ^]^ In one report, a postsynthetic film treatment was developed to increase the efficiency of the LED device through decreasing the number of crystalline surface defects in in situ formed MAPbI_3_ NC thin films.^[^ ^422^ ^]^ Methylammonium iodide was first added to the film in order to complete all of the surface octahedral structures, followed by the addition of bulky octylammonium iodides. This led to an enhanced device (ITO/poly‐TPD/MAPbI_3_ NCs/TPBi/LiF/Al) EQE up to ≈15%.^[^ ^422^ ^]^

#### 6.1.3 LHP NCs as Photoexcited Downshifting Emitters in WLEDs

Integrating LHP NCs as photoexcited downshifting emitters in LEDs and backlight displays represent another popular and effective way to utilize LHP NCs. WLEDs are commonly fabricated, and typically consist of a combination of LHP NC layers and other phosphor layers on top of a blue‐emitting LED chip. Typically, three layers are used, which emit in the blue, green, and red range, to produce an overall white light. In the case of these devices, a variety of concerns for the LHP NCs need to be taken into account to achieve efficient white light emission, including NC optical properties (such as strong absorption, high PL QY, etc.),^[^ ^91^, ^253^ ^]^ photo‐,^[^ ^218^, ^248^, ^253^, ^303^, ^316^ ^]^ color‐,^[^ ^218^, ^312^ ^]^ and thermal‐stabilities,^[^ ^97^, ^218^, ^248^ ^]^ and stabilities under ambient conditions.^[^ ^97^, ^302^, ^307^, ^312^ ^]^

**Figure 13:**
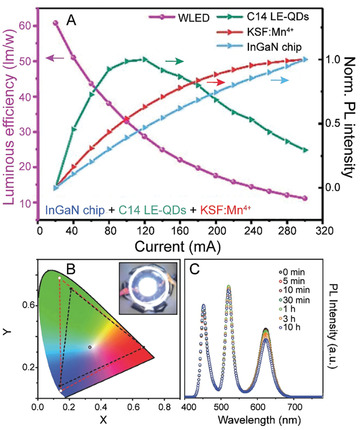
LHP NCs as photoexcited downshifting emitters in WLEDs. A) Luminous efficiency and PL intensity versus current for WLEDs fabricated with CsPbBr_3_ NCs that had undergone a ligand exchange with 1‐tetradecyl‐3‐methylimidazolium bromide. Adapted with permission.^[^ ^197^ ^]^ Copyright 2020, Elsevier. B) CIE color coordinates of a WLED device fabricated from a blue LED chip and green and red silica‐coated CsPbBr_3_ and CsPb(Br/I)_3_ NCs, respectively (white circle) and the color triangle of the WLED device (red dashed line) compared to the NTSC TV standard (black dashed line). The inset shows the fabricated WLED. C) The PL spectra of the WLED fabricated in (B) at different operation times demonstrating enhanced stability. B,C) Adapted with permission.^[^ ^218^ ^]^ Copyright 2016, Wiley‐VCH.

The first demonstration of the green‐emitting OA and OAm passivated MAPbBr_3_ NCs synthesized using the LARP procedure were utilized in the fabrication of WLEDs.^[^ ^45^ ^]^ These NCs were combined with a red‐emitting K_2_SiF_6_:Mn^4+^ layer on a blue‐emitting GaN chip.^[^ ^45^ ^]^ The resulting WLED demonstrated a luminous efficiency of 48 LuMens per Watt (Lm W^−1^) at a current of 4.9  mA and a color coordinate value of (0.33, 0.27) (equal energy white light has a color coordinate of (0.333, 0.333)) in the commission internationale de l’éclairage (CIE) chromaticity diagram (130% NTSC standard).^[^ ^45^ ^]^ LHP NCs have also been treated with DDAB ligands to achieve better NC passivation and enhance device stabilities.^[^ ^253^ ^]^ For example, devices fabricated using 100  µL of a 0.02 m DDAB solution, could maintain 80% of the initial PL intensity for more than 50 h, while devices fabricated without DDAB (only CsPbBr_3_ NCs) dropped to 50% of their PL intensity following 6 h of operation.^[^ ^253^ ^]^ Other, more complicated ligands like 1‐tetradecyl‐3‐methylimidazolium have been used to passivate CsPbBr_3_ NCs utilized in WLEDs through ligand exchange strategies.^[^ ^197^ ^]^ These LEDs demonstrated superior luminous efficiency (61.0 Lm W^−1^) compared to the devices fabricated from CsPbBr_3_ NCs that had not undergone the ligand exchange (**Figure** **13**A).^[^ ^197^ ^]^ Furthermore, the saturation current was increased from 80  mA for devices fabricated using native‐ligand CsPbBr_3_ NCs to 120  mA for the 1‐tetradecyl‐3‐methylimidazolium‐capped CsPbBr_3_ NCs (Figure 13A).^[^ ^197^ ^]^ These device performance improvements were attributed to the enhanced phosphor stabilities afforded from the ligand exchange.^[^ ^197^ ^]^

##### Encapsulated LHP NCs for Enhanced WLED Stability

Since stability is a major concern when fabricating LED devices, a variety of the encapsulation strategies which afforded LHP NCs enhanced stabilities were utilized to prepare the NCs for incorporation into WLEDs. For example, there are many reports of using LHP NCs coated with a silica shell for WLED fabrication.^[^ ^218^, ^224^, ^301^, ^302^, ^303^ ^]^ In one report, silica coated green emitting CsPbBr_3_ NCs were combined with silica coated red emitting CsPbBr*_x_*I_3−_ *_x_* NCs onto a blue LED.^[^ ^218^ ^]^ The silica coating prevented against anion exchange, allowing for enhanced color stability, and the ability for direct mixing of different LHP NC phosphors in the fabrication of the WLEDs.^[^ ^218^ ^]^ The best fabricated WLED showed a luminous efficiency of 61.2 Lm W^−1^, a CIE coordinate of (0.33, 0.33), and encompassed 120% of the NTSC standard (Figure 13B).^[^ ^218^ ^]^ Device stability was monitored by measuring the WLED emission spectrum upon increasing working times (Figure 13C).^[^ ^218^ ^]^ Specifically, no change in the emission spectrum was observed following 3 h of working time, but the device suffered from a decrease in intensity over prolonged periods of time for the red‐emitting CsPbBr*_x_*I_3−_ *_x_* NCs, indicating their less‐than ideal stabilities.^[^ ^218^ ^]^

Polymer coated LHP NCs have also been heavily utilized in the fabrication of WLED devices.^[^ ^95^, ^229^, ^307^, ^424^ ^]^ For example, polyimide‐coated CsPbBr_3_ NCs were synthesized using a continuous stir tank reactor and incorporated into a WLED along with blue emitting BaMgAl_10_O_17_:Eu^2+^ and red emitting (Sr,Ca)AlSiN_3_:Eu^2+^ phosphor layers on a UV chip (365  nm).^[^ ^226^ ^]^ Applying a driving current of 20  mA, the WLED possessed a CIE chromaticity coordinate of (0.361, 0.342) (135% NTSC space) and a color rendering index of 83.4.^[^ ^226^ ^]^ The devices fabricated with the polymer‐coated NCs also exhibited better operational stability than those fabricated with pristine NCs.^[^ ^226^ ^]^ LHP NCs fabricated using star‐like diblock copolymers also exhibited great optical properties, giving them high potential for incorporation into WLED devices.^[^ ^97^ ^]^ In one example, green‐emitting PS‐capped CsPbBr_3_ NCs and red emitting conventional CdSe/Cd_1−_ *_x_*Zn*_x_*Se_1−_ *_y_*S*_y_*/ZnS QDs were used to fabricate a WLED on a blue GaN chip.^[^ ^97^ ^]^ The resulting WLEDs had a CIE color coordinate of (0.31, 0.32) (130% NTSC standard).^[^ ^97^ ^]^ The device also exhibited remarkable stability, as it withstood storage for 400 days and exhibited 96% PL retention during a thermal cycle from 25–100  °C.^[^ ^97^ ^]^

##### Templated LHP NCs for Enhanced WLED Stability

WLED devices using the LHP NCs grown in silica, polymer and zeolite templates or matrices have also been demonstrated. For example, both CsPbBr_3_ and CsPb(Br_0.4_I_0.6_)_3_ NCs were added to a silicone resin (Dow Corning OE‐6631 A&B Kit) and then dropped on a blue chip to form a WLED device.^[^ ^306^ ^]^ Initially, the emission wavelengths of the two NC phosphor layers were correspondingly at 515 and 625  nm, then slowly red‐shifted and blue‐shifted, respectively, due to an anion exchange reaction of NCs in the silicone resin.^[^ ^306^ ^]^ To alleviate this issue, the CsPbBr_3_ NCs were first embedded into mesoporous silica, then added to the red‐emitting NCs in the silicone resin.^[^ ^306^ ^]^ Following this modification, no spectrum shifting was observed, and white light emission with a CIE coordinate of (0.24, 0.28) was achieved with a luminous efficiency of 30 Lm W^−1^.^[^ ^306^ ^]^ Besides silica matrices, LHP NCs have been embedded in large polymer networks or Zeolite templates before incorporation into WLEDs.^[^ ^228^, ^243^, ^248^, ^305^, ^312^, ^316^ ^]^ For example, MAPbX_3_ NCs embedded in a poly vinylidene fluoride matrix were used to form WLEDs (MAPbBr_3_ NC‐composite films, K_2_SiF_6_:Mn^4+^, InGaN chips) with a luminous efficiency of 109 Lm W^−1^ and a color gamut that was 121% of the NTSC standards.^[^ ^245^ ^]^ CsPbBr_3_ NCs embedded in Zeolite Y have been mixed with CsPb(Br_0.4_I_0.6_)_3_ NCs on a InGaN LED chip.^[^ ^248^ ^]^ The obtained WLED exhibited a CIE chromaticity coordinate of (0.38, 0.37) (114% NTSC standard).^[^ ^248^ ^]^ Zeolite embedding helped to increase the color output stability, which is typically affected due to poor thermal stability and photostability of LHP NCs upon exposure to the heat and light generated from the blue LED chip.^[^ ^248^ ^]^ Specifically, the luminous efficiency (at a diode current of 20  mA) for the LEDs with and without zeolite was 3.5 Lm W^−1^ versus 2 Lm W^−1^.^[^ ^248^ ^]^ Thus, while the zeolite‐embedded NCs were still found to degrade under strong and prolonged illumination, it was to a lesser extent than the pristine LHP NCs.^[^ ^248^ ^]^

### 6.2 Integration of LHP NCs in Photovoltaics

Since the first example of an LHP solar cell shown in 2009, where an LHP film was formed on TiO_2_ and the resulting device had a power conversion efficiency (PCE) of 3.8%,^[^ ^425^ ^]^ many groups have been working toward improving LHP‐based solar cell performances. While there have been numerous reports on utilizing LHP thin films as absorbers in photovoltaic devices,^[^ ^48^, ^426^, ^427^, ^428^, ^429^, ^430^ ^]^ increasing attention has been given to LHP materials on the nanoscale.^[^ ^414^ ^]^ There are now many excellent examples of LHP‐NC based solar cells where ligand engineering has been explored as a method to improve PCEs, which is the focus of the discussion in this section.

#### 6.2.1 Improvement of LHP NC‐Based Photovoltaics through Control over Ligand Densities and Identities

**Figure 14:**
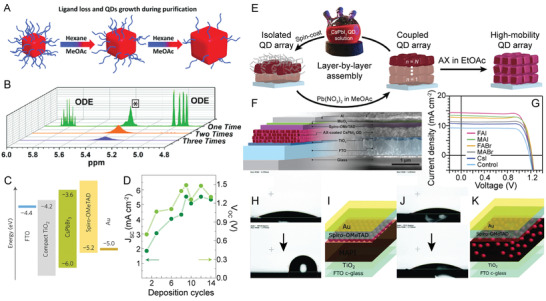
LHP NCs in solar cell devices. A) Schematic image of the changes in NC size and ligand coverage during purification treatments. B) Portion of the ^1^H NMR spectrum of CsPbBrI_2_ NCs during purification treatments shown in (A) exhibiting a decrease in ligand concentration. A,B) Reproduced with permission.^[^ ^432^ ^]^ Copyright 2020, Wiley‐VCH. C) Energy level diagram of materials used in a solar cell fabricated using a short‐chain ligand‐passivated (propionic acid and butylamine) CsPbBr_3_ NC ink. D) Short circuit current and open‐circuit voltage versus number of deposition cycles of CsPbBr_3_ NC ink for the solar cell device structure shown in (C) Adapted with permission.^[^ ^371^ ^]^ Copyright 2017, Nature Publishing Group. E) Schematic illustration of the CsPbI_3_ NC film formation process and AX salt treatment for solar cell device fabrication. F) Schematic cross section of a CsPbI_3_ NC‐sensitized solar cell overlaid on an SEM image of the cross‐section of the device. G) *J*–*V* characteristics of CsPbI_3_ NC solar cell devices treated with FAI (pink), MAI (green), FABr (yellow), MABr (gray), CsI (dark blue), and a no‐additive control (light blue). E‐G) Adapted with permission.^[^ ^436^ ^]^ Copyright 2017, AAAS. H) Contact angle measurement of a perovskite film without (above) and with (below) MAPbI_3_ NCs added to the top of the perovskite film. I) Schematic image of a solar cell device fabricated by adding MAPbI_3_ NCs to the top of the perovskite absorber layer. J) Contact angle measurement of a perovskite film without (above) and with (below) MAPbI_3_ NCs embedded within the film. K) Schematic image of a solar cell device fabricated by embedding MAPbI_3_ NCs into the perovskite absorber layer. H‐K) Reproduced with permission.^[^ ^439^ ^]^ Copyright 2018, Royal Society of Chemistry.

Similar to the studies previously discussed for LED applications, purification of the NCs to control ligand densities has also been related to solar cell device performance. For example, a PCE of 8.38% could be achieved by controlling ligand density through two washing steps using 2‐pentanol followed by acetonitrile for FAPbI_3_ NCs in the colloidal solution and an additional surface washing treatment during the film fabrication process with ethyl acetate.^[^ ^431^ ^]^ Additionally, current density‐voltage curves showed improvements in open circuit voltage (*V*_OC_), short circuit current (*J*_SC_), fill factor (FF), and PCE with each round of NC purification.^[^ ^431^ ^]^ In another example, methyl acetate could be used during purification to lower the ligand density and also resulted in a slight increase in NC size (**Figure** **14**A).^[^ ^432^ ^]^ The final ligand density on the NCs was determined after the first and second washing steps through quantitative ^1^H NMR measurements (Figure 14B).^[^ ^432^ ^]^ As such, the calculated ligand concentrations on the NCs with one‐, two‐ and three‐rounds purification were 2.35, 1.87, and 1.01 wt%, respectively.^[^ ^432^ ^]^ These three NC samples were incorporated into solar cells, and the PCE was found to increase upon decrease in ligand shell density (from 5.9% for NCs with a ligand density of 2.35 wt%, to 12.2% for NCs with a ligand density of 1.01 wt%).^[^ ^432^ ^]^

NC purification and ligand density also affect behaviors at the interface between the LHP absorber layer and the hole‐ or electron‐transport layers. In one study, the effects of post synthetic LHP NC treatments on the dynamics of charge separation was studied using LHP NC–TiO_2_ composite materials, where both the photoinduced electron transfer from the NCs to the TiO_2_ and the charge recombination from the TiO_2_ to the NCs were explored.^[^ ^433^ ^]^ After loss of ligand density following washing procedures, the charge separation rates decreased, which corresponds to an increase in charge separation efficiency.^[^ ^433^ ^]^

In addition to ligand density, identity of the ligands passivating the LHP NCs used for photovoltaic applications is also very important for device performances. A slow charge recombination process is typically required in order to minimize the competition with separating excited electrons in the conduction band (CB) and holes in the valence band (VB).^[^ ^404^ ^]^ As such, a commonly explored method to improve photovoltaic solar cells is through enhancing charge separation and transport capabilities. One way to accomplish this is through the use of short chain ligands.^[^ ^371^, ^373^ ^]^ For example, short chain propionic acid and butylamine were used as ligands to form CsPbBr_3_ nanoinks.^[^ ^371^ ^]^ These nanoinks were then incorporated into solar cells (fluorine doped tin oxide (FTO)/TiO_2_/CsPbBr_3_ NCs/2,2,7,7‐tetrakis‐(*N*,*N*‐*p* dimethoxyphenylamino)‐9‐spirobifluorene (Spiro‐MeOTAD)/Au) through a spin coating technique (Figure 14C).^[^ ^37^ ^]^ Following one layer of spin coating, the device had a *J*_SC_ of 1.26  mA cm^−2^, a *V*_OC_ of 0.87  V and a FF of 0.65, resulting in a PCE of 0.72%.^[^ ^371^ ^]^ The low photocurrent for this device, however, was attributed to the thin NC layer. In response, multiple layers were sequentially spin coated onto the device, facilitated by the low boiling point solvents and ligands of the nanoinks.^[^ ^371^ ^]^ Increasing the film thickness resulted in a monotonic growth of optical density along with an increase in both *J*_SC_ and *V*_OC_, consequently leading to a maximum PCE of 5.4% (Figure 14D).^[^ ^371^ ^]^ The effects of shorter‐carbon chain ligands on NC film quality led to a further study where these NC films were analyzed following thermal annealing,^[^ ^373^ ^]^ a strategy oftentimes required in device fabrications. Annealing these NC films resulted in ligand loss, sintering and transformation to form micro CsPbBr_3_ and tetragonal CsPb_2_Br_5_ phase materials.^[^ ^373^ ^]^ In another example, CsPbI_3_ NCs were used as the absorber layer through a spray‐coating technique.^[^ ^434^ ^]^ However, the final device displayed poor performances (PCE of 0.96%), which were attributed to the insulating nature of the ligand chains (i.e., OA and OAm).^[^ ^434^ ^]^ To solve this issue, the authors treated each layer that was spray‐coated with Pb(NO_3_)_2_ in methyl acetate to remove excess ligands, which boosted the PCE to a much higher value of 6.8%.^[^ ^434^ ^]^ The performance of the solar cell device was further improved by passivating the NCs with short carbon‐chain ligand, i.e., phenyltrimethylammonium bromide, to increase carrier mobility, resulting in a PCE of 11.2%^[^ ^434^ ^]^

#### 6.2.2 LHP NC Thin Film Improvement for Enhanced Solar Cell Device Performances

One of the simplest ways to improve solar cell device efficiency is through careful NC film post processing. In one example, CsPbI_3_ NC films were dipped into a methyl acetate solution that was saturated with either lead acetate or lead nitrate.^[^ ^435^ ^]^ This treatment aided in film stability, suppressing the transition to a nonperovskite phase, and enhanced the PL, leading toward a PCE of 10.77%.^[^ ^435^ ^]^ Similarly, perovskite A‐site cation halide (AX) salts (i.e., CsX, MAX, FAX) can be used to coat NC arrays and have been shown to improve electron coupling in CsPbI_3_ NC films (Figure 14E).^[^ ^436^ ^]^ A variety of AX salts have been tested in the fabrication of solar cells (FTO/TiO_2_/AX‐coated CsPbI_3_ NCs/Spiro‐OMeTAD/MoO*_x_*/Al) (Figure 14F), leading to enhancements in device performances, as shown in the *J*–*V* scans (Figure 14G).^[^ ^436^ ^]^ A maximum PCE of 13.4% was achieved when a device was treated with FAI.^[^ ^436^ ^]^ Addition of ligands commonly used in NC syntheses, such as OA, has also been shown to improve film stability in solar cell devices, resulting in better performances after being aged in 76% humidity for four weeks.^[^ ^437^ ^]^

#### 6.2.3 Integrating LHP NCs into LHP Thin Films

Another strategy to improve solar cell performance is through the integration of LHP NCs into bulk‐scale LHP thin films. In one example, CsPbX_3_ NCs were deposited onto bulk LHP films in order to get improved hole injection and electron blocking characteristics at the interface between the LHP layer and the hole transport material (i.e., Spiro‐MeOTAD).^[^ ^379^ ^]^ In another study, CsPbBr_3_ NCs were added to the surface of a perovskite film, along with a conductive diammonium porphyrin, to create a perovskite capping layer based on porphyrin‐bridged NCs.^[^ ^438^ ^]^ This capping layer led to efficient charge transport and separation, resulting in an enhanced PCE reaching 20%.^[^ ^438^ ^]^ The device also showed improved thermostability at 85  °C (maintaining over 65% of the PCE values following 1000 h of operation) and superior operational stability (maintaining over 85% of the PCE values following exposure to 45% humidity at 25  °C for 1000 h).^[^ ^438^ ^]^

Beyond improvements of charge carrier separation/transport processes, OA and OAm passivated MAPbI_3_ NCs have been incorporated into the absorber perovskite layer of solar cells to improve the hydrophobicity of the device which can be crucial for operational stability.^[^ ^439^ ^]^ Device architectures with LHP NCs deposited on top of LHP thin films (Figure 14I) were hydrophobic, with water contact angles higher than 100° (Figure 14H, bottom), which was a great improvement from the films fabricated without the NCs (Figure 14H, top).^[^ ^439^ ^]^ On the other hand, devices fabricated with LHP NCs incorporated throughout the LHP thin film (Figure 14K) did not improve the contact angle (Figure 14J), demonstrating the need for LHP NCs, with their hydrophobic ligands, on the surface of the film.^[^ ^439^ ^]^ Beyond improving the hydrophobicity of the solar cell devices, the photovoltaic performance was tested for the LHP NC‐containing films.^[^ ^439^ ^]^ The most efficient solar cell possessed the LHP NCs deposited on top of the LHP film (Figure 14I), and gave a *V*_OC_ of 0.74, a *J*_SC_ of 20.89  mA cm^−2^, a FF of 68.52% and a PCE of 10.6%.^[^ ^439^ ^]^ Devices with MAPbI_3_ NCs embedded in the perovskite layers showed much lower PCEs, which was attributed to misalignment in band edge positions.^[^ ^439^ ^]^

### 6.3 LHP NCs in Other Devices and Applications

#### 6.3.1 LHP NC‐Based Photodetectors and Scintillators

Due to their excellent optical performances including high PL QYs, narrow tunable emissions, and long carrier diffusion lengths, LHP NCs have been explored in multiple optoelectronic applications.^[^ ^37^, ^38^, ^43^, ^46^, ^47^, ^48^, ^49^, ^50^, ^51^, ^52^, ^53^, ^54^, ^55^ ^]^ Beyond the previously discussed LED and solar cell devices, incorporation of LHP NCs into photodetectors has also been a prominent area of research.^[^ ^384^, ^385^, ^386^, ^440^, ^441^, ^442^ ^]^ Similar to other optoelectronic devices, charge transfer property of the applied materials is crucial for fabrications of high‐quality photodetectors. As such, there have been many studies focused on engineering LHP NC ligands and supports for enhanced charge transfer.^[^ ^386^, ^387^, ^442^, ^443^ ^]^ In one study, CsPbX_3_ NCs capped with OA were directly used in the fabrication of a photodetector, however, charge transport was not ideal due to the insulating OA ligands, even after annealing procedures.^[^ ^387^ ^]^ To improve the charge transport property, mesoporous TiO_2_ was utilized as a scaffold which resulted in a twofold PL reduction, indicative of the efficient charge transfer from the CsPbX_3_ NCs to the TiO_2_.^[^ ^387^ ^]^ Consequently, the devices with the TiO_2_ scaffold demonstrated 44‐times enhanced photoresponsivity compared to the devices prepared without TiO_2_.^[^ ^387^ ^]^ Ligands have also been utilized to form a bridge between LHP NCs and TiO_2_.^[^ ^386^ ^]^ For example, 3‐mercaptopropionic acid (MPA) was used as a bifunctional linker ligand to attach CsPbX_3_ NCs with TiO_2_ nanoparticles.^[^ ^386^ ^]^ Specifically, the thiol group on MPA could interact with the CsPbX_3_ NCs while the carboxylic group on MPA could interact with the hydroxyl groups on the surface of the TiO_2_ nanoparticles.^[^ ^386^ ^]^ With this linking design, the photodetector exhibited a superior responsivity and detectivity and the total electron transfer time from the CsPbX_3_ NCs to the TiO_2_ particles was shorter than if the two were in direct contact (40  ns vs 290  ns).^[^ ^386^ ^]^

**Figure 15:**
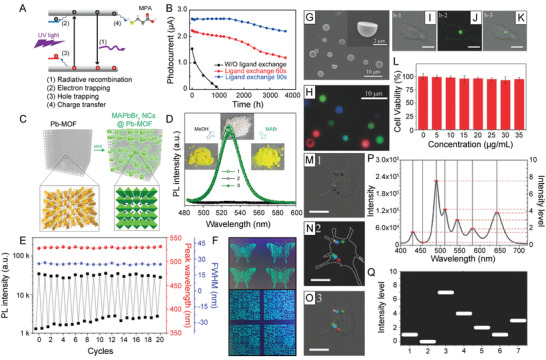
A) Schematic image of the competing dynamic relaxation processes of CsPbCl_3_ NCs passivated with 3‐mercaptopropionic acid (MPA) for photodetector applications. B) Photocurrent versus time for photodetectors without (black) and with (red = 60  s and blue = 90  s) ligand exchange with MPA. A,B) Adapted with permission.^[^ ^442^ ^]^ Copyright 2019, American Chemical Society. C) Schematic image of the formation of MAPbBr_3_ NCs in a Pb‐MOF matrix through the addition of MABr for use in encryption/decryption technologies. D) PL spectra demonstrating the on/off fluorescence of the MAPbBr_3_ NC @ Pb‐MOF composites through the addition of methanol (off) and MABr (on). E) PL intensity, peak position and FWHM of the fluorescent emission of MAPbBr_3_ NC @ Pb‐MOF composites versus on/off cycle number. F) Printed QR code (top) and butterfly (bottom) using the MAPbBr_3_ NC @ Pb‐MOF composites. C‐F) Reproduced with permission.^[^ ^391^ ^]^ Copyright 2017, Nature Publishing Group. G) Low magnification SEM image of CsPbX_3_ NC @ microhemisphere composites to serve as luminescent probes in cell imaging. Inset shows a higher resolution SEM image of a single CsPbX_3_ NC @ microhemisphere composite. H) Fluorescence image of the CsPbX_3_ NC @ microhemisphere composites with varying halide compositions. Bright‐field I), fluorescent J), and bright‐field and fluorescence overlay K) images of macrophage (RAW 264.7) cells incubated with CsPbBr_3_ NC @ microhemisphere composites. (Scale bars = 15  µm). L) Cell viability versus concentration of CsPbBr_3_ NCs in the microhemispheres. Bright‐field M), fluorescence N), and a bright‐field and fluorescence overlay O) images of live macrophage cells incubated with a mixture of mixed‐halide CsPbX_3_ @ microhemispheres. (Scale bars = 20  µm). P) PL spectrum of the multiplexed emission of the CsPbX_3_ NC @ microhemisphere composites. Q) Fluorescence 2D barcode for the PL spectrum shown in (P) for optical coding. G–Q) Adapted with permission.^[^ ^230^ ^]^ Copyright 2017, Wiley‐VCH.

Besides, MPA has worked as a chemical linker to improve LHP NC‐graphene interactions for photodetectors through completely quenching both irradiative recombination, and electron and hole trapping (**Figure** **15**A).^[^ ^442^ ^]^ The concentration of MPA ligands could be controlled by changing the amount of time utilized for the ligand exchange reaction, and the photoresponse time dramatically decreased from 192.8 to 0.25 s for NCs without MPA ligand passivation and NCs that underwent MPA ligand exchange for 120 s, respectively.^[^ ^442^ ^]^ Additionally, the photocurrent increased from 1.54  µA for NCs passivated with native ligands to 2.67  µA for NCs after 90 s of ligand exchange (Figure 15B).^[^ ^442^ ^]^ A study was also done to understand charge transport quality of CsPbI_3_ NC/reduced graphene oxide (rGO) heterostructures without MPA.^[^ ^443^ ^]^ The use of rGO as a scaffold allowed for the NC synthesis to proceed with lower concentrations of ligands, as the rGO separated the NCs and prevented against aggregation.^[^ ^443^ ^]^ This NC/rGO heterostructure composite gave the potential to exhibit enhanced conductivity, carrier transport quality and other optoelectronic properties.^[^ ^443^ ^]^ The superior carrier separation and transport were demonstrated by a lengthened PL LTs compared to CsPbI_3_ NCs, which implied longer carrier diffusion lengths.^[^ ^443^ ^]^ Finally, the cyclic voltammetry (*C*–*V*) curve of the heterostructures showed that there were no clear oxidation or reduction reaction voltages, demonstrating that the direct growth of the NCs on rGO can alter the carrier transport behavior, likely through charge injection into the rGO layer.^[^ ^443^ ^]^

In addition to the rGO scaffold, CsPbBr_3_ NCs have been formed on MoS_2_ monolayers to study device performance as a function of charge transfer at the interface between the two materials.^[^ ^441^ ^]^ Specifically, under repeated operating conditions, the photodetector performance is determined by both the absorption capability of the LHP NCs as well as the charge transfer efficiency from the LHP NCs to the MoS_2_ monolayer.^[^ ^441^ ^]^ Increasing the concentration of LHP NCs can boost the device absorption capabilities, and proper ligand content (OAm/OA) can enhance interfacial exciton dissociation and charge transfer.^[^ ^441^ ^]^ In terms of ligands specifically, the effects of purification cycles on device performances were explored.^[^ ^441^ ^]^ As the number of purification cycles increased, the concentration of ligands were lowered, resulting in NC aggregation and a decrease in the device currents and photoresponsivities.^[^ ^441^ ^]^ Decrease in ligand concentration also led to an increase in surface defects, leading to reduced PL LTs and reduced charge transfer.^[^ ^441^ ^]^ Thus, a balance between surface defect passivation and charge transfer is needed to achieve better device performances.^[^ ^441^ ^]^

Beyond utilizing UV and visible light excitation to achieve LHP NC PL, inorganic CsPbX_3_ LHP NCs and nanosheets have been demonstrated in scintillating applications, where X‐ray radiation could be converted to emitted photons in the visible region.^[^ ^382^, ^444^, ^445^, ^446^ ^]^ To explore new scintillation material candidates, heavy atoms are required as X‐ray absorption scales with effective atomic number (*Z*_eff_).^[^ ^382^, ^447^ ^]^ As such, inorganic, Pb^2+^‐containing LHP NCs hold the promise and have been utilized as lower‐cost materials for scintillation with a superior X‐ray detection capability.^[^ ^382^, ^444^ ^]^ In the first reported example, oleate‐capped CsPbX_3_ NCs were used to achieve multicolor emission in scintillator devices fabricated through the deposition of the NCs onto flexible substrates.^[^ ^382^ ^]^ The fabricated devices were sensitive even at low doses of 5.0  μGy s^−1^ at 10  kV and 5  µA, making them comparable to high efficiency CsI:Tl bulk scintillators.^[^ ^382^ ^]^

PL QY of the LHP NCs is an important factor to increase the sensitivity of scintillator devices.^[^ ^446^ ^]^ For example, LHP nanosheets were used to form high quality scintillators in both colloidal and solid forms.^[^ ^446^ ^]^ Specifically, the nanosheets were able to self‐assemble into large area films through face‐to‐face alignment with an inter‐nanosheet gap of 2.1  nm owing to the presence of two layers of ligands (octylamine or octanoic acid).^[^ ^446^ ^]^ The films retained high PL QYs following assembly due to the retainment of the ligands which were shielded during the self‐assembly process.^[^ ^446^ ^]^ The fabricated scintillator devices demonstrated strong radioluminescence and reduced radio‐degradation effects following continuous X‐ray illumination for 2 h.^[^ ^446^ ^]^

#### 6.3.2 LHP NCs in Lasing Applications

LHP NCs have also demonstrated high potential for lasing applications due to their high PL QYs, and the ability to compositionally tune their PL properties.^[^ ^448^ ^]^ As such, LHP NCs have been utilized as optical gain medium for lasing applications, where amplified spontaneous emission (ASE) has been observed using low pump thresholds.^[^ ^51^, ^448^, ^449^ ^]^ Inorganic CsPbX_3_ nanocubes capped with OA and OAm have been demonstrated in lasing applications, all resulting in low threshold ASE (pump thresholds as low as 5  µJ cm^−2^).^[^ ^51^, ^380^, ^449^ ^]^ CsPbBr_3_ nanocubes have also been formed in inorganic, low‐melting TeO_2_‐based glasses without the use of any organic ligands.^[^ ^450^ ^]^ The glass enabled superior NC stability and the homogeneous distribution of the nanocubes within the glass offered the composite great potential as a disorder gain medium for upconversion lasing.^[^ ^450^ ^]^

Other NC morphologies, such as nanorods, nanowires, and nanoplatelets, have been utilized to serve as more effective gain media and/or to form natural resonant cavities.^[^ ^451^, ^452^, ^453^, ^454^, ^455^ ^]^ While many of these reports employed synthetic methods, such as chemical vapor deposition^[^ ^451^, ^452^, ^454^ ^]^ and reprecipitation techniques,^[^ ^453^ ^]^ that did not utilize ligands, some of the studies specifically exploited ligands to control the NC morphology.^[^ ^455^ ^]^ Specifically, alteration of the ratio between OA and OAm used in a LARP synthesis of FAPbX_3_ NCs led to the formation of nanospheres or nanoplatelets, both of which could be used for two‐photon pumped ASE.^[^ ^455^ ^]^ While the nanoplatelets could realize controllable lasing by themselves, nanospheres suffered from strong scattering loss and weak optical feedback.^[^ ^455^ ^]^ To alleviate these issues, external cavities, such as microcapillary tubes, were explored.^[^ ^455^ ^]^ In more detail, the differences in refractive index between the microcapillary tubes and the NCs works to confine the NC PL, inducing whispering‐gallery mode lasing (PL travels around concave microcapillary surface).^[^ ^455^ ^]^ Chemically coupling LHP NCs to silicon dioxide microspheres is another strategy that has been used to achieve whispering‐gallery mode lasing.^[^ ^456^ ^]^ In this case, the silica microspheres are first aminated by (3‐aminopropyl)triethoxysilane and then added to the LHP NC precursors in the hot injection synthesis.^[^ ^456^ ^]^ The NCs are then able to connect to the surface of the microspheres during synthesis, through the surface‐exposed amine functional groups.^[^ ^456^ ^]^ The silica microspheres then function as a cavity during lasing, achieving a lasing threshold of 750  µJ cm^−2^.^[^ ^456^ ^]^

#### 6.3.3 LHP NCs in Encryption and Decryption, Anticounterfeiting, and Detection

The PL properties of LHP NCs have been used in forming secret messages for encryption and decryption and anticounterfeiting applications. In one example, sequential templated growth and dissolution of MAPbX_3_ NCs in a Pb‐MOF template was demonstrated for encryption and decryption applications.^[^ ^391^ ^]^ Specifically, Pb‐based MOFs were used both as a Pb^2+^ source and as a template for forming emissive MAPbX_3_ NCs when exposed to MAX (Figure 15C).^[^ ^391^ ^]^ The emission could then be subsequently quenched through dissolution of the LHP NCs through treatment with polar solvents (i.e., methanol) (Figure 15D).^[^ ^391^ ^]^ Numerous cycles of this formation/dissolution treatment could be repeated, continually resulting in strong PL upon NC formation with negligible decrease in PL intensity after 20 cycles, rendering the system useful to encrypt and decrypt secret messages (Figure 15E).^[^ ^391^ ^]^ For instance, complicated images and QR codes were invisibly printed using a Pb‐MOF ink, and the images could be revealed following treatment with MAX salt (Figure 15F).^[^ ^391^ ^]^

Similar to encryption and decryption, LHP NC materials have been utilized to print patterns that could be used in anticounterfeiting applications.^[^ ^223^ ^]^ Specifically, CsPbBr_3_@Cs_4_PbBr_6_/SiO_2_ composites were used due to their high stability (imparted from Cs_4_PbBr_6_ and SiO_2_), excellent optical properties (imparted from CsPbBr_3_), and triple‐modal (UV, IR, thermal) characteristics.^[^ ^223^ ^]^ Furthermore, the composites were readily dispersible in polydimethylsiloxane silica gel to form inks for spin‐coating, spraying, or inkjet printing.^[^ ^223^ ^]^ Fluorescence of the printed patterns could be turned on through illumination with UV light (325  nm) and IR light (800  nm, through two photon absorption) and off through halting illumination or by heating the films to 150  °C, providing multiple ways to reveal or hide the NC patterns.^[^ ^223^ ^]^

Beyond their applications in printing patterns for secret messages or anticounterfeiting applications, the excellent fluorescent properties of LHP NCs have also been used for detection of different chemical species in solutions.^[^ ^196^ ^]^ In one example, water‐dispersible 2D PEA_2_PbI_4_ (PEA = phenethylammonium) NCs could be synthesized by using higher concentrations of PEA^+^ in order to prevent ligand desorption and favor stability.^[^ ^196^ ^]^ Due to their dispersibility in water, the NCs could be utilized to detect Cu^2+^ in an aqueous environment, which has relevance in biological systems.^[^ ^196^ ^]^ Specifically, the PL intensity of the NCs was found to decrease upon increasing concentrations of Cu^2+^ in solution, demonstrating the material's potential in detection applications.^[^ ^196^ ^]^ In another example, MAPbBr_3_ NCs were used for the selective detection of explosive 2,4,6‐trinitrophenol (TNP).^[^ ^392^ ^]^ Specifically, octylammonium bromide‐capped NCs were found to detect as low as femtomolar (10^−15^ m) concentrations of TNP through monitoring fluorescence quenching in solution, and even showed quenching when exposed to TNP vapor.^[^ ^392^ ^]^ The NCs were unable to detect other nitroaromatics tested, such as 2,4‐dinitrotoluene and 2,4,6‐trinitrotoluene, showing great selectivity for TNP.^[^ ^392^ ^]^ This selectivity was attributed to interaction of the TNP with the NCs through hydrogen bonding.^[^ ^392^ ^]^

#### 6.3.4 LHP NCs as Fluorescent Probes for Sensing and Biomedical Imaging

Encapsulated LHP NCs that demonstrate stability in aqueous environments have been utilized in bioimaging applications. As discussed above in Sections  3.4.3 and  4.2.2, PS is a commonly used LHP NC encapsulation polymer that has shown great promise for biological applications.^[^ ^230^, ^316^ ^]^ Specifically, CsPbX_3_ NCs coated with PS have demonstrated good stability in water, acid aqueous solutions, alkali aqueous solutions, phosphate buffer solution, etc.^[^ ^316^ ^]^ Furthermore, CsPbBr_3_ NC@PS composites have exhibited strong green PL following nine months storage in water.^[^ ^316^ ^]^ Due to these excellent stabilities, PVP‐capped LHP NCs embedded into PS microhemispheres were incubated with macrophage (RAW 264.7) cells and resulted in internalization of the CsPbX_3_ NC@microhemisphere composites for macrophage labeling (Figure 15G–K).^[^ ^230^ ^]^ Cell viability remained ≈100% after 24 h following incubation with microhemispheres containing increasing concentrations of CsPbX_3_ NCs, demonstrating the durability of the microhemisphere coating (Figure 15L).^[^ ^230^ ^]^ The fluorescent NC@microphemisphere composite probes themselves could be precisely tuned, through alteration of both halide composition and PL intensity (Figure 15H).^[^ ^230^ ^]^ Specifically, seven unique PL emissions could be accessed through changing halide composition, resulting in unique PL emissions without spectral overlap.^[^ ^230^ ^]^ Furthermore, PL intensity of an individual composite could be altered by adjusting the loading density of the CsPbX_3_ NCs in the microhemispheres.^[^ ^230^ ^]^ Introduction of these varying probes into a single macrophage cell (Figure 15M–O) gave a signature PL emission spectrum (Figure 15P) which could be translated into a 2D “barcode” (Figure 15Q) for precise cell labeling, making it possible for individually tagging ≈10 million cells.^[^ ^230^ ^]^ In another example, CsPbBr_3_ NCs embedded in PS were incubated with HeLa cells, and also showed low cytotoxic effect, even at concentrations as high as 400  µg mL^−1^.^[^ ^316^ ^]^ However, following incubation at 37  °C for 1 h, green PL was only observed around the edge of the cells, as the composites were difficult to internalize due to their larger sizes (>400  nm) and hydrophobic interactions between PS and the phospholipid molecules in the cell membrane.^[^ ^316^ ^]^ Phospholipid micelle encapsulated CsPbX_3_ NCs have also been incubated with HeLa cells, achieving multi‐colored bioimaging within 5 min.^[^ ^309^ ^]^ These composites had a positive surface potential, facilitating endocytosis and entry of the biomarkers into the cytoplasm.^[^ ^309^ ^]^

Beyond simple encapsulation of LHP NCs for biological applications, CsPbX_3_ NCs have been co‐encapsulated with magnetite in PVP to form water‐resistant magneto‐fluorescent nanocomposites.^[^ ^310^ ^]^ The composites were transfected into MDA‐MB‐231 mammospheres to demonstrate their use in bioimaging and cell tagging.^[^ ^310^ ^]^ Specifically, they exhibited strong fluorescence in the cells and did not photobleach over time.^[^ ^310^ ^]^ Furthermore, the cells were attracted to an external magnet due to the presence of magnetite within the composites.^[^ ^310^ ^]^ LHP NCs have also been used in biological imaging and tracking through adding additional functionality to the encapsulation shell.^[^ ^315^ ^]^ In one example, Anti‐CD63 antibodies were incorporated onto the surface of CsPbBr_3_ NCs coated with block copolymers through noncovalent interactions to detect and track triple negative breast cancer cell lines.^[^ ^315^ ^]^ These composites could successfully bind to the surface of the MDA‐MB‐231 breast tumor‐derived exosomes, while lacking binding to HaCaT cells without overexpressed CD63 proteins, demonstrating specificity.^[^ ^315^ ^]^

## 7 Conclusion

The rapid development of LHP NCs in response to their superior optical and optoelectronic properties has shown promise for their future applications. As playing multiple critical roles in different aspects of LHP NCs, advancing the knowledge in ligand shell development is critical and inevitable to push these materials forward. In this regard, challenges and opportunities coexist, which represent active research areas that still require significant efforts of development. For example, the tri‐component LHP crystal structure complicates surface‐ligand interactions, requiring additional considerations that are not required for conventional semiconducting QD NCs. In addition to exploring new passivating and functional ligands, novel ligand combinations and inter‐ligand interactions on the LHP NC surfaces should be paid more attention, which may bring new insights and controllability of the materials. Besides, inorganic and/or small molecule ligands deserve to be more carefully investigated for LHP NCs, especially when surface accessibility or charge separation and transfer are the highly desired metrics for applications such as photocatalysis, photodetector and solar cell devices.^[^ ^10^, ^457^, ^458^ ^]^ Uniquely, ligands of LHP NCs have been demonstrated to invoke crystal structural transformations. This opens the door toward precise, postsynthetic structural control, which can be potentially expanded to utilize other ligand triggers and/or lead‐free halide perovskite‐systems. On that note, as the field is quickly evolving, lead‐free perovskite NC systems are becoming more prevalent due to the exclusion of toxic lead from the crystal structure and improved particle and crystal stabilities.^[^ ^65^, ^327^, ^330^, ^459^, ^460^, ^461^, ^462^, ^463^ ^]^ Insights gained from the ligand studies of LHP NC systems should be, in principle, largely transferable to lead‐free counter parts due to their common surface ions (e.g., Cs, halides). Furthermore, for the encapsulation strategies, compatibility studies of the encapsulating materials with understanding long‐term effects of the final composite constructs on desired properties are needed to predict life cycles of LHP NC‐composite containing devices. Meanwhile, the new particle surfaces that are exposed following encapsulation should not be overlooked. Finally, understandings of ligand behavior and fate in LHP NC‐based solids have been still underdeveloped and will be beneficial to further optimize and ready these materials for solid‐state applications, and ultimately position LHP NCs for related commercialization. In all, given the unparalleled development history of the field and continuously increased attention from research disciplines, we have no doubt that the delineated challenges will be overcome, and the pledged opportunities will be realized.

## Conflict of Interest

The authors declare no conflict of interest.
